# Cooking methods affect advanced glycation end products and lipid profiles: A randomized cross-over study in healthy subjects

**DOI:** 10.1016/j.xcrm.2025.102091

**Published:** 2025-04-24

**Authors:** Judith Wellens, Eva Vissers, Anaïs Dumoulin, Sien Hoekx, Julie Vanderstappen, Joke Verbeke, Roman Vangoitsenhoven, Muriel Derrien, Bram Verstockt, Marc Ferrante, Christophe Matthys, Jeroen Raes, Kristin Verbeke, Séverine Vermeire, João Sabino

**Affiliations:** 1 Department of Chronic Diseases and Metabolism, Translational Research Center for Gastrointestinal Disorders (TARGID), KU Leuven, 3000 Leuven, Belgium; 2 Department of Gastroenterology and Hepatology, University Hospitals Leuven, 3000 Leuven, Belgium; 3 Department of Chronic Diseases and Metabolism, Clinical and Experimental Endocrinology, KU Leuven, 3000 Leuven, Belgium; 4 Department of Endocrinology, University Hospitals Leuven, 3000 Leuven, Belgium; 5 Microbiology, Immunology and Transplantation Department, Rega Institute, KU Leuven, 3000 Leuven, Belgium; 6 VIB Center for Microbiology, 3000 Leuven, Belgium

**Keywords:** advanced glycation end products, carboxymethyl lysine, CML, MG-H1, pyrraline, cooking methods, culinary techniques, 4E-BP1, butyric acid, dietary intervention

## Abstract

Thermal treatments used in ultra-processed foods (UPFs) lead to advanced glycation end products (AGEs). UPFs and serum AGEs are associated with cardiometabolic disease. We explore differential cooking methods as a mechanistic link between UPFs and detrimental health outcomes through a randomized cross-over cooking method trial in healthy subjects using identical ingredients and a deep profiling analysis. We show that low-AGE-generating cooking methods such as boiling and steaming decrease serum AGEs, improve lipid profiles, and increase serum protein 4E-BP1. In contrast, high-AGE-generating cooking methods such as grilling and baking increase fecal butyrate. In sum, this suggests that low-AGE-generating cooking methods should be considered in cardiovascular risk prevention. Since current dietary guidelines focus on ingredients, but not cooking methods, our results suggest that culinary techniques should be considered as an important factor in cardiometabolic preventive strategies and future dietary trial design. This study was registered at ClinicalTrials.gov (NCT06547190).

## Highlights

• Cooking methods alter food’s biological impact, even with identical ingredients
• Boiling and steaming improve lipid profiles and increase serum protein 4E-BP1
• Grilling and baking increase advanced glycation end products and fecal butyrate
• Culinary techniques should be considered in cardiometabolic preventive strategies

## Graphical abstract

**Figure:**
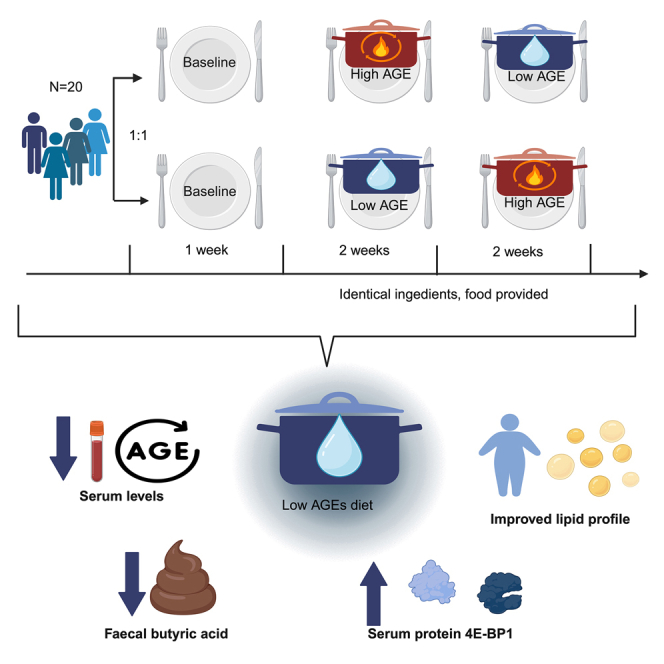

## Teaser

Wellens et al. explore the effects of cooking methods that generate differential levels of advanced glycation end products (AGEs) in food on human health. They suggest that low-AGE-generating cooking methods should be considered in cardiovascular prevention strategies and dietary trial design.

## Introduction

Ultra-processed food (UPF) consumption has increased dramatically and in parallel with a variety of chronic diseases.^1^^,^^2^^,^^3^ Although epidemiological evidence linking exposure to outcome is growing, specific causal mechanisms remain unknown. To improve flavor, texture, shelf life, and biological safety of food products, thermal treatments are a method of choice for the food industry.^4^ Upon heating, intermediate and reversible Maillard reaction products are formed, which can stabilize into dietary advanced glycation end products (dAGEs).^5^^,^^6^^,^^7^ Cooking methods like boiling and steaming generate low amounts of advanced glycation end products (AGEs), while baking and grilling produce higher amounts due to higher temperatures and dry heat.^6^

Carboxymethyl lysine (CML) as well as pyrraline and methylglyoxal are major dAGEs in common foods and are therefore widely used as indicators of the dAGE level.^6^^,^^8^ In humans, AGEs lead to cross-linking of proteins thereby altering their structure and function and, by interaction with AGE-binding receptors, initiating an inflammatory cascade and generating oxidative stress.^6^^,^^9^ Non-absorbed AGEs can theoretically modulate gut microbial composition and function or bind to receptors at the colonic epithelium, although data on the effect of dAGEs on gut microbial composition, metabolism, and inflammation in human remain scarce.^10^^,^^11^^,^^12^^,^^13^ In addition, human trials aimed at reducing dAGEs claim an improvement of cardiometabolic parameters through increased insulin sensitivity, lowering of low-density lipoprotein (LDL) cholesterol, and reduced systemic inflammatory markers and oxidative stress.^14^^,^^15^^,^^16^^,^^17^ Yet, inconsistencies in current literature remain, probably due to uncontrolled study designs, lack of comparable macronutrient intake, and differing study populations.^15^ In addition, although grilling produces high amounts of AGEs, this cooking technique is currently promoted by dietary guidelines because it necessitates little amounts of oils and fats.^18^ However, longitudinal studies have shown that high levels of serum AGEs are independently associated with increased risk of cardiovascular disease.^19^ To date, many studies focus on culinary techniques without adequately controlling for macronutrient and micronutrient intake. However, it was recently shown that assessment of both food products and cooking techniques was necessary to predict serum AGE levels.^20^ Therefore, to fully understand the role of dAGEs in preventive medicine, understanding the effect of cooking methods is a necessary step that is currently underexplored.

Here, we performed a well-controlled cross-over randomized study in healthy individuals and focused on the effect of varying AGE levels produced solely by altering cooking methods using a deep profiling approach that encompasses systemic and intestinal outcomes.

### Study overview

**Figure 1:**
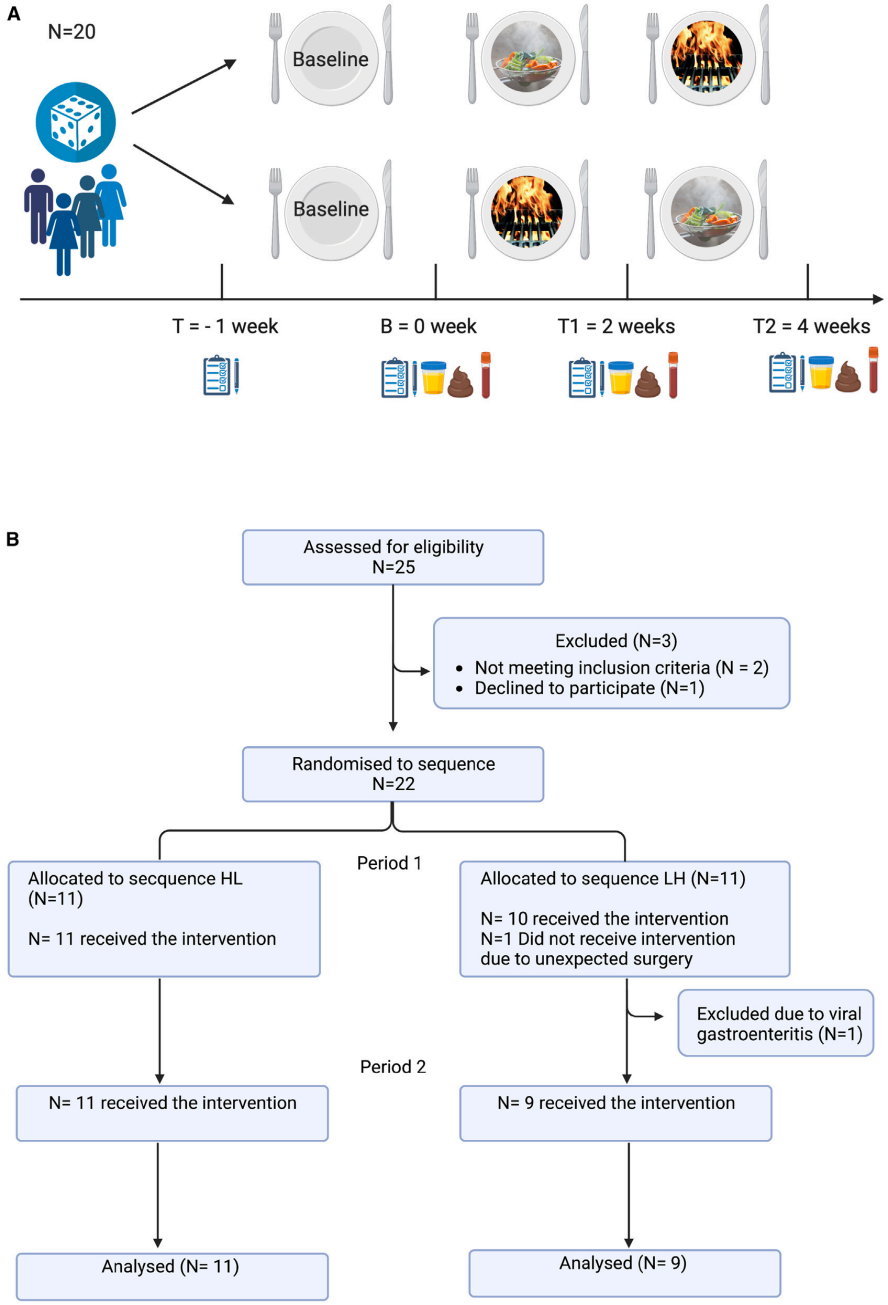
Study design and CONSORT flow diagram (A) Study design: open-label randomized cross-over controlled trial. Before and after each interventional period anthropometric measurement, blood, stool, and fecal samples were taken. (B) Consolidated Standards of Reporting Trials (CONSORT) flow diagram for randomized cross-over trials detailing flow of trial participants. T, time point; B, baseline; T1, time point 1; T2, time point 2; HL, high-to-low AGE diet; LH, low-to-high AGE diet.

Participants were randomly allocated to start with a low-to-high AGE diet (LH) or a high-to-low AGE diet (HL) based on different cooking instructions (Figure 1A). Each dietary intervention period consisted of two weeks after which the second dietary intervention period started. Both diets contained the same ingredients with recipes varying by cooking method to obtain different dAGE levels (Figure S1 and Table S1). The study diet consisted of a granola breakfast, a bread-based meal, a home-cooked meal, and snacks. All ingredients were provided by the study team. Food packages were available for take-out twice a week at a local supermarket, except for seasoning. A steam cooker and all recipes were provided. Alcohol consumption and smoking were not allowed during the trial. Anthropometric outcome parameters included weight, hand grip strength, and blood pressure measurements. AGE levels were calculated based on nutritional intake and measured in serum using mass spectrometry. Intestinal outcome measurements included fecal calprotectin, small intestinal permeability using the lactulose-mannitol ratio (LMR) and serum lipopolysaccharide-binding protein (LBP) ELISA, and assessment of the gut microbial composition using 16S rRNA sequencing and short-chain fatty acids (SCFAs) measurements. Systemic inflammation and metabolomic outcomes were assessed using Olink proteomics and routine laboratory testing.

## Results

### Cooking methods affect serum AGE levels

In this exploratory randomized cross-over trial in twenty healthy volunteers, 75% were female, the mean age was 32 (±12 years), and body mass index (BMI) was 23.5 (±3.6 kg/m^2^) (Table 1). All subjects were current non-smokers, with one participant having smoked minimally in the past. One participant withdrew before starting the trial due to unexpected surgery. One participant was requested to discontinue the study because of viral gastroenteritis during the first interventional period (Figure 1B). No harm occurred during the trial. Before the intervention, most participants usually consumed home-cooked meals, and the most frequently used cooking methods included frying, boiling, and using microwave and oven (Figure S2).

**Table 1:** Baseline demographic characteristics, *n* = 20

Characteristic	Median ± IQR or frequency (%)	Range (minimum–maximum)
**All (*n* = 20)**		

Age (years)	28 (23.5–38)	19–59
BMI (kg/m^2^)	23.01 (20.9–25.4)	19.6–31.9
Gender (female)	15/20 (75%)	–
Smoking status	19 never smoked (95%)	–
	1 ex-smoker (5%)	–
**HL (*n* = 11)**		

Age (years)	28 (26–38)	19–59
BMI (kg/m^2^)	24.40 (23.1–25.9)	19.6–31.9
Gender (female)	6/11 (54.6%)	–
Smoking status	10 never smoked (90.91%)	–
	1 ex-smoker (9.09%)	–
**LH (*n* = 9)**		

Age (years)	26 (23.3–45.5)	21–51
BMI (kg/m^2^)	22.3 (21.0–23.2)	20.1–27.0
Gender (female)	9/9 (100%)	–
Smoking status	9 never smoked (100%)	–

Data are represented as median ± interquartile range (IQR) or frequency (%).

**Figure 2:**
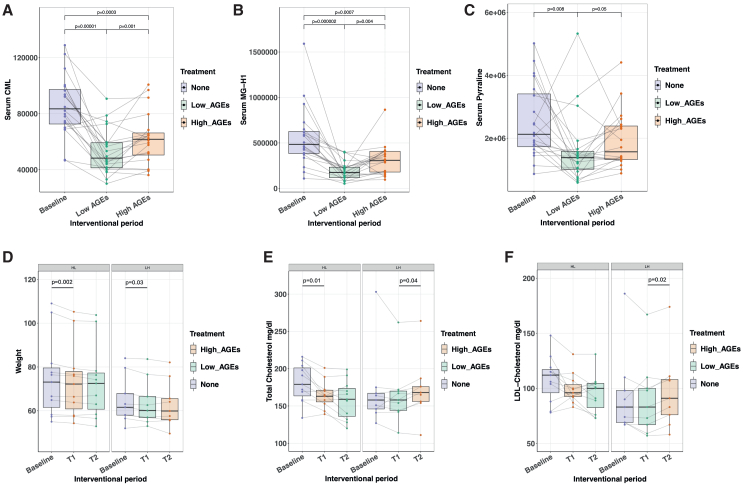
Serum advanced glycation end products, weight, and cholesterol (A–C) Serum advanced glycation end products measured by mass spectrometry. Samples were taken at baseline, labeled as “None,” and after each intervention, labeled as low-AGEs or high-AGEs depending on the intervention that was received. (A) carboxymethyl lysine (CML). (B) N-(5-hydro-5-methyl-4-imidazolon-2-yl)-ornithine (MG-H1). (C) pyrraline. (D) Weight in kg. (E) Total cholesterol levels (mg/dL). (F) LDL cholesterol levels (mg/dL). T1, time point 1; T2, time point 2. Wilcoxon signed rank test. HL, high-to-low AGE diet; LH, low-to-high AGE diet. Data are represented as mean ± SD.

Compared to the recipes from the high-AGE-generating cooking methods, there was an average 2-fold decrease in estimated CML content when using low-AGE-generating cooking methods (*p* = 0.0001, Wilcoxon rank-sum test, Table S2). Serum concentrations of the AGEs CML and N-(5-hydro-5-methyl-4-imidazolon-2-yl)-ornithine (MG-H1) were significantly lower after the low-AGE diet than after the high-AGE diet (*p* = 0.001 and *p* = 0.004, respectively, Wilcoxon signed rank test), with pyrraline showing a trend in the same direction (*p* = 0.05, Wilcoxon signed rank test, Figures 2A–2C and Table S3). Quantified serum AGE levels were markedly higher at baseline compared to the high-AGE diet, unlike estimated CML content through the food logs (Table S3).

### Stable macronutrient composition between interventions

Participants were instructed to eat *ad libitum* from the provided ingredients and recipes. Yet, for the full cohort during the whole trial, we report a significant mean weight loss of 1.8 kg (SD 1.6 kg, Wilcoxon signed rank test, *p* = 0.0005, Table S4). Most of the weight loss was driven by participants with a higher BMI at the start of the study and after the first interventional period (Figure 2D) (mean weight loss of 1.2 kg, SD 1.2, Wilcoxon signed rank test, *p* = 0.0005 after the first period and 0.6 kg, SD 1.1, *p* = 0.03 after the second period).

One participant was excluded from dietary analyses due to incomplete food record reporting, meaning failure of daily registration of the food log. A similar energy intake (kcal) between the two interventional periods was observed (Wilcoxon signed rank *p* > 0.1), which was also similar to baseline energy intake (Wilcoxon signed rank, *p* = 0.07 compared to high-AGE diet and *p* = 0.1 compared to the low-AGEs diet). When comparing the two interventional periods, energy, fat, carbohydrate, protein, and fiber intake were similar (Wilcoxon signed rank test, *p* > 0.1). However, compared to baseline, carbohydrate intake increased (Wilcoxon signed rank test *p* = 7.6 × 10^−6^ for the low-AGE and Wilcoxon signed rank test *p* = 3.8∗10^−5^ for the high-AGE period compared to baseline), while protein and fiber intake followed the inverse pattern. Protein intake significantly increased during the low-AGE period (*p* = 0.001) and the high-AGE period (*p* = 0.002). Likewise, fiber intake significantly increased during the interventional period compared to baseline (Wilcoxon signed rank test, baseline vs. low-AGE diet *p* = 0.004 and baseline vs. high-AGE diet *p* = 0.05). There were no significant differences in fat and salt intake between baseline and the intervention (Wilcoxon signed rank test, *p* > 0.1, Figures S3A–S3E and Tables S5A–S5F). Overall physical activity level in metabolic equivalent minutes per week was not different between any of the study periods (Wilcoxon signed rank test, *p* > 0.1, Figure S4 and Tables S6A and S6B).

### Cooking methods affect lipid profiles

Total cholesterol and triglycerides decreased in the full cohort after the first interventional study period (mean decrease of 11 mg/dL, SD 16, Wilcoxon signed rank test, *p* = 0007 for total cholesterol and a mean decrease of 18 mg/dL, SD 21, *p* = 0.001 for triglycerides, Table S4). Interestingly however, total cholesterol values increased significantly with a mean of 8 mg/dL, SD 8 (Wilcoxon signed rank test, *p* = 0.04, Figures 2E and 2F), after consuming the high-AGE diet in the LH group. Moreover, when the HL group went from the high-AGE to the low-AGE study diet, total cholesterol values decreased, although non-significantly with 7 mg/dL, SD 13 (Wilcoxon signed rank test, *p* > 0.1). These findings were equally seen for high-density lipoprotein (HDL) cholesterol measurements and to a lesser extent for LDL cholesterol and triglycerides, together suggesting a beneficial effect of the low-AGE diet on the lipid profile.

### A diet low in AGEs affects the serum proteome

Inflammation, as reflected by serum C-reactive protein (CRP) concentrations, significantly decreased after the first interventional period, irrespective of cooking method (Wilcoxon signed rank test, *p* = 0.01). Fecal calprotectin concentrations remained stable (Wilcoxon signed rank test, *p* > 0.1), yet, in two distinct participants, an increase >200 μg/g was noted after the high-AGE diet, which returned to baseline after completing the low-AGE diet. Interestingly, both at baseline and at the end of the trial, their fecal calprotectin levels were <30 μg/g.

**Figure 3:**
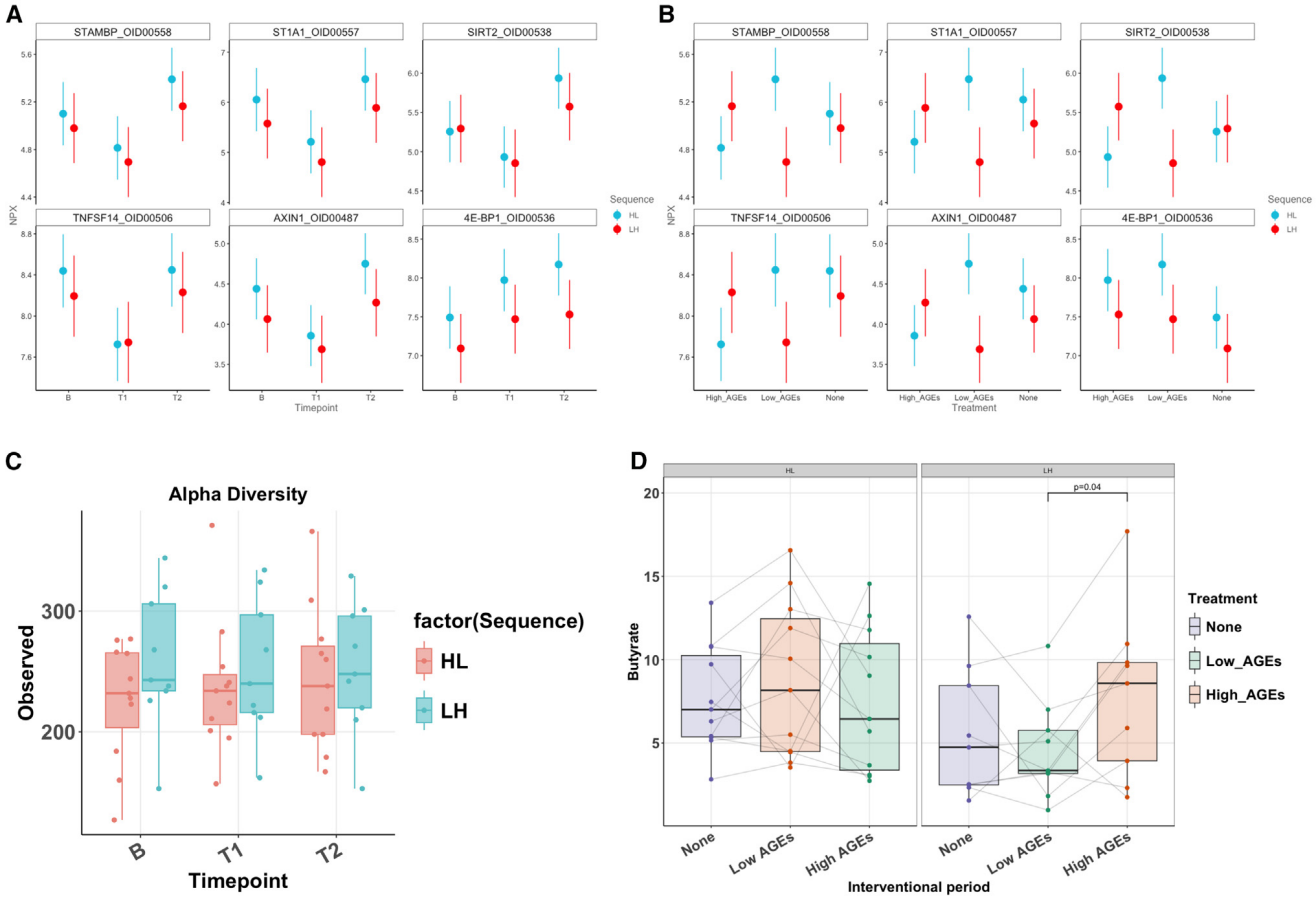
Host proteomics and gut microbiota (A and B) Results of serum inflammatory and cardiometabolic proteomic profiles using a linear mixed model per protein. (A) Time point. (B) Treatment. (C) Alpha diversity at baseline, after the first interventional period (T1) and after the second interventional period (T2) per sequence. (D) Results of the fecal butyrate at every time point (baseline, after the first interventional period [T1], and after the second interventional period [T2]) per sequence, concentration in microgram/100 mg feces. Data are represented as mean ± SD.

Next, we performed a deep profiling of immune and metabolic profiles using 184 serum markers. Different cooking methods did not impact the overall serum inflammatory or cardiometabolic proteomic profiles (permutational multivariate analysis of variance [PERMANOVA] *p* > 0.1, Figures S5A–S5C). However, the dietary shift compared to the habitual diet introduced by the intervention did influence the serum inflammatory proteomic profile as depicted in the principal-components analysis plot (PERMANOVA *p* = 0.002, Figure S5B). In the linear mixed model, STAM binding protein (STAMBP), Sulfotransferase Family 1A Member 1 (ST1A1), Sirtuin 2 (SIRT2), Tumor necrosis factor superfamily member 14 (TNFSF14), and AXIN1 were significantly associated with time point (all adjusted *p* values < 0.05). These five proteins decreased after the first two weeks of any dietary intervention, and a rebound effect was observed in the following two weeks, irrespective of the group (Figures 3A and 3B). Only eukaryotic initiation factor 4E-binding protein (4E-BP1) was significantly associated with the dietary intervention.

### A diet high in AGEs increases gut microbial butyrate production

There were no differences seen in alpha diversity between groups, nor between time points using observed, Simpson, inverse Simpson, and Shannon diversity indices (all *p* > 0.1, Wilcoxon signed ranked test, Figure 3C). Likewise, beta diversity (Bray-Curtis’s dissimilarity index) remained stable (*p* > 0.1, Wilcoxon signed rank test). When analyzing the intra- and interindividual Bray-Curtis’s dissimilarity dynamics, the dietary treatment again did not significantly affect the overall gut microbial composition (both *p* > 0.1, Wilcoxon signed rank test, Figure S6A). Similarly, there were no differences in abundances at the genus level that survived multiple testing corrections. Overall microbiota composition (principal coordinates analysis [PCoA]) revealed differences between the habitual diet and any of the experimental diets (Adonis2 function stratified by individual, baseline vs. T1: *p* = 0.007 and baseline vs. T2: *p* = 0.01). However, no changes were seen between the high-AGE and the low-AGE diets (pairwise Adonis, stratified by individual, *p* > 0.1), suggesting that the observed changes were due to a change in dietary habits, rather than a change in cooking methods (Figures S6B and S6C). In addition, PCoA revealed a separation between sequences (LH and HL, *p* = 0.008, Envfit) and gender (*p* = 0.005). Additional variation in the PCoA was mainly explained by BMI (*p* = 0.001, Envfit), handgrip strength (*p* = 0.003), waist-hip ratio (*p* = 0.01), hemoglobin (*p* = 0.001), CRP (*p* = 0.004), total cholesterol (*p* = 0.005), fasting glucose (*p* = 0.02), and Bristol Stool score (*p* = 0.006), but not a change in serum AGEs (Figure S6D).

When looking at gut microbial metabolites by analyzing SCFAs, a significant increase in fecal butyrate was measured after the high-AGE study period in the LH group (Wilcoxon signed rank test, *p* = 0.04, Figure 3), which seemed to be mirrored, although not statistically significant, in the HL group. There were no changes in fecal acetate nor propionate.

### Dietary intervention alters small bowel intestinal permeability

Paracellular intestinal permeability was assessed using a functional sugar test (fractional urinary LMR), and transcellular permeability was quantified through LBP in serum. Compared to baseline, the LMR was increased in the HL group after consuming the high-AGE diet (*p* = 0.02, Wilcoxon signed rank test) and the low-AGE diet, both compared to baseline (*p* = 0.001, Wilcoxon signed rank test) (Figure S7A). In the LH group, there was a similar trend toward a higher intestinal permeability (Wilcoxon signed rank test baseline vs. T1 *p* = 0.08; baseline vs. T2 *p* = 0.1). In contrast, LBP showed no difference between groups nor between interventions (Figure S7B). Since the reported effect on LMR was not coupled to a specific intervention, this could be explained by the altered diet, rather than by differential cooking methods.

## Discussion

Here, we present a randomized cross-over cooking intervention study in healthy individuals that focused on systemic and intestinal outcomes, using a deep profiling approach.

We show that an intervention using differential cooking methods, while using the exact same ingredients, significantly alters serum AGEs. In addition, we confirmed that a low-AGE diet has a beneficial effect on the lipid profile.^15^

Both high serum AGEs and cholesterol levels have independently and longitudinally shown to be associated with increased risk of cardiovascular disease.^15^^,^^19^^,^^21^^,^^22^ However, current dietary guidelines do not consider cooking methods as a means of disease prevention except to reduce fat intake (e.g., grilling necessitates use of less fats) or in terms of food safety regarding pathogen control.^18^^,^^23^^,^^24^^,^^25^ Our findings suggest that culinary techniques can alter serum AGE levels independent of the macro nutritional composition and challenge current dietary recommendations. The statistically significant drop of circulating AGEs by almost 50% after a dietary intervention is striking as previous research suggests that dAGEs only minimally contribute to total serum AGEs as only 10%–30% are thought to be absorbed.^26^

Regarding intestinal permeability, we found no changes that were coupled to a cooking method. Similarly, we found no significant changes in intestinal inflammation nor in gut microbial composition associated with differential cooking methods either. However, and perhaps surprisingly given the negative gut microbial results, we did observe an increase in fecal butyrate after consumption of the high-AGE diet, which might indicate a change in gut microbial metabolism or a decrease in uptake of butyrate. Indeed, previous studies using preclinical models showed similar findings. In a batch-type simulator model using human microbiota, after incubation of the fecal material with glycated or unglycated pea protein, increased concentrations of acetate, propionate, and butyrate were seen in the former condition, together with increased levels of lactobacilli and bifidobacteria.^27^ In rats, an increased butyrate production was observed when fed a glycated fish protein diet as opposed to a non-glycated fish protein diet.^28^ Taking these observations one step further, exposure to CML and highly heated foods, respectively, alleviated gut microbial dysbiosis and even colitis in dextran sulfate sodium salt-treated mice, which is a frequently used animal model for inflammatory bowel diseases, suggesting that increased fecal butyrate after an high-AGE diet is beneficial to gut health.^29^^,^^30^ However, since this increase in fecal butyrate was not paralleled by an increase in butyrate producers, nor other SCFAs or changes in fecal calprotectin, our results do not necessarily point toward increased butyrate production. An alternative explanation to increased fecal levels of butyrate might be due to decreased colonic uptake. Indeed, butyrate has been shown to inhibit the interaction between the receptor for AGEs (RAGE) and their ligand, i.e., RAGE-ligand interaction.^31^ Through this interaction, we could hypothesize that full butyric acid absorption is impaired, yet the intestinal tissue remains relatively spared from downstream effects of RAGE including oxidative stress and even tumorigenesis. Clearly, further research is necessary to confirm our findings and understand potential interactions between butyrate and gut health in relation to RAGE.

Next, we found that the 4E-BP1 serum protein was significantly increased after consumption of the low-AGE diet. 4E-BP1 is a protein encoded by the *Eukaryotic translation initiation factor 4E-binding protein 1* gene, which is a substrate of the mechanistic target of rapamycin (mTOR).^32^ mTOR is dependent on environmental inputs such as nutrients and growth factors and coordinates many fundamental processes in eukaryotic cell growth and metabolism.^33^ Dysregulation of mTOR signaling is implicated in the progression of cancer, diabetes, and the aging process.^33^^,^^34^ Murine data suggest that overexpression of 4E-BP1 has positive effects on health span as it protected male mice from diet-induced obesity and insulin resistance.^35^^,^^36^ On the other hand, since mTOR inhibitors are also actively studied to promote lifespan and in cancer treatments, the clinical relevance of this finding in our study is unclear at the moment and would require further study.

In summary, we conducted a deep-profiling analysis in healthy adults to investigate the effect of differential cooking methods on intestinal and metabolic health.

We found that, when using the exact same ingredients, altering cooking methods significantly alters serum AGE levels after a two-week intervention. Next, a diet low in AGEs improved the lipid profile and increased serum levels of the protein 4E-BP1 but had no effect on other cardiometabolic or inflammatory markers related to the cooking method. We also found that a diet high in AGEs increased fecal butyrate concentrations levels.

Our results question current dietary guidelines on lifestyle and cooking advice. Given the effect of cooking methods on host serum markers and imperfect calculations based on food frequency questionnaires (FFQs) alone, culinary techniques merit attention when developing diet-based interventions in the prevention and treatment of chronic metabolic diseases.

### Limitations of the study

We acknowledge certain limitations. As this was an exploratory feeding study, the duration of the intervention was relatively short, which may have prevented further delineation of the effect of the studied diets. In addition, our sample size is rather small, and 75% of our participants were female; however unfortunate, females are typically overrepresented in the fields of preventive medicine, endocrinology, and nutritional and metabolic sciences.^37^ However, we were able to leverage the sample size and reduce interindividual variation through the cross-over design. We did, however, opted to not implement a washout period as we showed that the baseline diet of healthy volunteers is high in AGEs, which would therefore also lead to carryover. In addition, as the macro nutritional composition might also affect results, returning to a habitual diet during a washout period seemed rather unattractive. However, using our design, we cannot rule out the presence of any carryover effects.

Second, in dietary studies, adherence is critical yet difficult to achieve, and participants might under- or misreport dietary intake.^38^ To mitigate this, all food products were provided by the study team, and all participants were asked to take pictures of their meals to ascertain consumption of the right products using the appropriate cooking method. Unfortunately, due to strict adherence to the low-calorie-dense foods during the trial, a statistically significant weight loss was noted, likely due to increased attention to diet and improvement in overall dietary quality, keeping in mind that alcohol and soda consumption were not allowed during the trial and that fiber intake indeed increased, which might affect our results.

Third, we observed differences between the estimated and measured serum AGE contents, which could be due to the fact that only 10%–30% of dAGEs are expected to be absorbed.^10^ In addition, the consulted database of Uribarri et al. is commonly used yet includes American products, which might lead to inaccuracies in estimating the dAGE content of our Belgian meals.^14^^,^^39^ However, it was recently shown that knowledge on cooking methods was necessary to predict serum AGE levels on top of the calculated levels derived from FFQs and using the same database.^20^ This underscores the importance of knowledge of culinary techniques or direct serum measurement when assessing AGEs in human trials. Among these lines, it is also noteworthy that for two out of three measured serum AGEs the levels were higher after the baseline diet than after the high-AGE diet, which probably dilutes the observed effects between the study diets. Potential causes include the consumption of different food products and cooking methods such as deep-frying during baseline, but not during the interventional part of the study, to keep the macronutrient composition during the intervention as similar as possible. It remains therefore interesting to also compare each intervention to baseline to have a more complete picture of the findings.

Lastly, it should be noted that thermal heating of products also generates possible mutagenic and carcinogenic Maillard reaction products such as heterocyclic amines and acrylamides and might destroy antioxidants and heat-labile vitamins.^10^^,^^40^ However, participants were instructed not to blacken their food while cooking. It is noteworthy that, by varying cooking methods, food texture and food matrices can vary, which can impact the availability of micronutrients, which we were not able to account for. Nonetheless, since both diets were balanced and designed by registered dieticians, minimal requirements should have been met and any differences between groups are not expected to have a direct or fast effect on the outcome parameters after any interventional period.

## Resource availability

### Lead contact

Any further information or requests should be directed to and will be fulfilled by the lead contact, João Sabino (joao.sabino@uzleuven.be).

### Materials availability

This study did not generate new unique reagents.

### Data and code availability

• Participant-level clinical data are deposited to REDCap database (managed by Leuven University Hospital) and can be accessed via the lead contact by reasonable request.
• No custom statistical computer coding was used in this paper. Any used code or additional information required to reanalyze the data reported in this work paper is available from the lead contact upon request.
• The Olink dataset can be accessed in the PRIDE database under the accession number PRIDE: PXD059624, while the 16S microbiota dataset is available in the ENA database under the accession number ENA: PRJEB83973. Any additional information required to reanalyze the data reported in this work paper is available from the lead contact upon request.

## Acknowledgments

We would like to acknowledge the Collect&Go service from Colruyt Group Heverlee, Belgium, for facilitating this research. We want to thank the collaborators of the VIB Metabolomics Core Leuven and all participants for their valuable contribution. We also thank Ken De Smet for drawing all blood samples. We would also like to thank all lab technicians (Sophie Organ, Tamara Coopmans, Helene Blevi, Kirsten Rems, and Hannelore Hoogsteyn), our lab manager Arno Cuvry from the IBD group, and the lab technician Greet Vermeulen at TARGID, KU Leuven, for processing the samples. Figures were created with Biorender.com and using icons from Flaticon.

J.W. was supported by the Flemish Crohn’s and Ulcerative Colitis patient organization (Crohn & Colitis ulcerosa Vereniging vzw, CCV) research grant (2021). The 10.13039/501100003130Research Foundation – Flanders (Fonds Wetenschappelijk onderzoek [FWO]), Belgium, supported J.W. (1S06023N), J. Verbeke (1S96721N), and E.V. (1SH1L24N) with a PhD Fellowship strategic basic research (SB) grant. M.F. and J.S. are supported by a Senior Clinical researcher grant from 10.13039/501100003130FWO. B.V. is supported by the Clinical Research Fund (KOOR) at the University Hospitals Leuven and the 10.13039/501100004497Research Council, KU Leuven.

## Author contributions

Study conceptualization and design: J.S. and J.W. Participant recruitment and running the trial: J.W. Sample collection: J.W., E.V., S.H., and J. Vanderstappen. Anthropometric measurements: S.H., J. Vanderstappen, and J.W. Validation of FFQ: J. Verbeke. Formal data analysis: J.S., J.W., and A.D. Interpretation of the data: all authors. First draft: J.W. Final draft editing and approval: all authors.

## Declaration of interests

The authors declare no competing interests.

## STAR★Methods

### Key resources table

**Table:** 

REAGENT or RESOURCE	SOURCE	IDENTIFIER
**Biological samples**		

Serum samples	This study	N/A
Fecal samples	This study	N/A
Urine samples	This study	N/A

**Critical commercial assays**		

Human LBP ELISA Kit	Invitrogen, USA	Cat#: EH297RBX10
Olink® Target 96 Inflammation	Uppsala, Sweden	Cat#: 95302
Olink® Target 96 Cardiometabolic	Uppsala, Sweden	Cat#: 95360
fCAL ELISA Kituo	Bühlmann	Cat#: EK-CAL

**Deposited Data**		

16S sequencting data - microbiota	This study	European Nucleotide Archive (ENA), accession number ENA: PRJEB83973
Olink proteomics data	This study	ProteomeXchange (PRIDE), accession number PRIDE: PXD059624

**Software and algorithms**		

R studio, software version 4.2.2	https://cran.r-project.org/https://www.rstudio.com	N/A
IBM SPSS Statistics, version 27	https://www.ibm.com/spss	N/A

### Experimental model and study participant details

#### Study design and setting

The Study on Treating by Eating: role of AGEs on Mucosal barrier and Microbiome (STEAMM) is an exploratory pilot parallel group, randomized cross-over trial in twenty healthy volunteers to assess the effect of different cooking methods on inflammation and metabolism. Because of the exploratory nature of the study, no formal sample size calculation was performed. All participants were recruited at the Leuven University and UZ Leuven through local advertisement and online employee media between February 1^st^ and May 3^rd^, 2022.

The study was approved by the Ethical Committee of the University Hospitals Leuven (S65600). All participants gave written informed consent prior to the start of the study and sample collection. ClinicalTrials.gov registration: NCT06547190.

#### Eligibility criteria

Healthy adults from age 18 to 65 without chronic illness were eligible to take part in the study. In addition, adhering to an omnivorous diet, Body Mass Index (BMI) between 18.5 and 30 kg/m^2^, and absence of an eating disorder were required. Participants with chronic illnesses were excluded including diabetes mellitus, cardiovascular disease, or cancer; eating disorders, irritable bowel syndrome; inflammatory bowel diseases, and previous bowel surgery (except for appendectomy). Current smokers were not eligible. Participants were not allowed to take nonsteroidal anti-inflammatory drugs (NSAIDS), proton pump inhibitors, antacids, or laxatives one month before enrollment or antibiotics, prebiotics or probiotics six months before the start of the trial. Due to slow recruitment, two participants with a BMI of >30 (but <32 kg/m^2^) were included as well.

#### Study participant details

Of the twenty included participants, 15 were of female gender, and the mean age was 32 (±12 years). Written informed consent was obtained before the start of the study. The study was approved and overseen by the Ethical Committee of the University Hospitals Leuven (S65600).

### Method details

#### Study intervention

Participants were randomly allocated to start with a low-to-high AGEs diet (LH), or a high-to-low AGEs diet (HL) based on different cooking instructions using R software using the randomizeR package.^41^ Neither participants nor researchers were blinded to the allocation. Each dietary intervention period consisted of two weeks after which the second dietary intervention period started. Both diets contained the same ingredients with recipes varying by cooking method to obtain different dAGE-levels. The study diet consisted of a granola breakfast, a bread-based meal, a home-cooked meal, and snacks. All ingredients were provided by the study team. Food packages were available for take-out twice a week at a local supermarket (Collect & Go service of Colruyt, Heverlee, Belgium), except for seasoning. A steam cooker (BRAUN, IDENTITY FS5100WH) and all recipes were provided. The granola was prepared and provided by the study team as well. Participants were allowed to drink water, mint infusions, milk, fruit juice and eat fresh fruits as well. Coffee and tea were not allowed during the trial due to the high dAGEs content. Alcohol consumption and smoking was not allowed during the trial.

#### Outcome measures

Anthropometric outcome parameters included weight, hand grip strength, and blood pressure measurements. Intestinal outcome measurements included fecal calprotectin, small intestinal permeability using the lactulose-mannitol ratio (LMR) and serum lipopolysaccharide binding protein (LBP) ELISA, and assessment of the gut microbial composition using 16S rRNA sequencing and short chain fatty acids (SCFA) measurements. Systemic inflammation and metabolomic outcomes were assessed using Olink proteomics and routine laboratory testing including hemoglobin, WBC count and differentiation, platelet count, urea, uric acid, serum creatinine, sodium, potassium, chloride, phosphate, calcium, magnesium, alkaline phosphatase, gamma-glutamyl transferase, bilirubin, total cholesterol, HDL-cholesterol, LDL-cholesterol, triglycerides, fasting glucose, and CRP. All outcomes were exploratory and assessed at all visits. There were no changes to the outcome measures after the trial had started.

#### Dietary intake assessment

At the start of the study, participants filled-in in a validated 32-item semi-quantitative Food Frequency Questionnaire (FFQ) as previously described.^42^ Baseline use of cooking methods was assessed using a questionnaire (Table S7). All participants were asked to keep a detailed daily food record starting a week before the first visit until the last day of the study through the smartphone application FatSecret. Participants were also requested to photograph each food product they consumed. Daily consumption of energy and nutrients was calculated per day by combining the information from the photographs as well as the food records. If inconsistencies arose, participants were directly contacted. Nutrients of the consumed food items were calculated using the Belgian Food Composition Table.^43^

#### Anthropometric measurements

Body weight was measured at each study visit, minimally dressed, to the nearest 0.1 kg on a digital scale. Abdominal (waist) and gluteal (hip) circumferences were taken according to the guidelines of the International Standards for Anthropometric Assessment and the waist to hip ratio (WHR) was calculated.^44^ Blood pressure was measured using a digital blood pressure monitor (Omron 5 series, Omron Healthcare, INC, Lake Forest, USA) in a seated position. Hand grip strength was measured using the hydraulic hand dynamometer (JAMAR). To assess the physical activity level in all participants, the short IPAQ was used at every visit.^45^

#### Quantification of lipopolysaccharide-binding protein (LBP) using enzyme-linked immunosorbent assays (ELISAs)

A Human LBP ELISA Kit (Invitrogen, USA) was used for the quantitative detection of serum LPS-binding protein according to the manufacturer’s instructions. All samples were run in triplicate and after in-house optimization, 1:500 dilutions were used.

#### Serum Advanced glycation end product assessment

The dAGE intake was estimated based on the previously published database of Uribarri et al.^6^ When data for certain cooking methods or ingredients were not available in the dataset, data obtained by a similar method or with a similar ingredient were used instead.

In addition, serum Advanced Glycation End products were also measured in serum using Mass Spectrometry. Twenty μL of serum was added to 180μL of extraction buffer (Methanol 80% containing 3 μM ^13^C_5_-D_5_-^15^N Glutamic acid, 3μM D7-^15^N_4_ Arginine, 1μM D_27_ myristic acid and 5μM D_12_ Glucose as internal standards) The samples were kept overnight at −80°C. Next, the samples were centrifuged at 20.000xg on a tabletop centrifuge for 15 min at 4°C. The supernatant was transferred to Mass Spectrometry (MS) vials for further analysis. Samples were analyzed using a Vanquish LC System (Thermo Scientific) coupled via heated electrospray ionization to a Q Exactive Orbitrap Focus mass spectrometer (Thermo Scientific). 10 μL sample was taken from an MS vial and injected onto a 15cm Poroshell 120 HILIC-Z PEEK Column (Agilent InfinityLab). A linear gradient was carried out starting with 90% solvent A (acetonitrile with 5μM medronic acid) and 10% solvent B (10 mM NH4-formate in Milli-Q water, pH 3.8). From 2 to 12 min the gradient changed to 60% B. The gradient was kept on 60% B for 3 min and followed by a decrease to 10% B. The chromatography was stopped at 25 min. The flow was kept constant at 0.25 mL/min and the column was kept at 25°C throughout the analysis. The HESI-source operated at positive polarity mode using a spray voltage of 3 kV, sheath gas at 45, auxiliary gas at 10, the latter heated to 260°C. The ion transfer capillary temperature was 320°C. The mass spectrometer operated in full scan (alternating between range [70.0000–1050.0000] to measure internal standards and range [190–290] to maximize sensitivity for the AGEs. The AGC target was set at 3.0E+006 using a resolution of 70000. Data collection was performed using the Xcalibur software (Thermo Scientific). The data analyses were performed by integrating the peak areas (El-Maven – Polly - Elucidata).

#### Serum proteomics

Cytokine data were generated from serum samples submitted to Olink Proteomics for analysis using the inflammation and cardiometabolic panel assay of 92 analytes (Olink INFLAMMATION and CARDIOMETABOLIC Target 96). All samples were shipped to Uppsala Science Park in Uppsala, Sweden and run by Olink. Data are presented as normalized protein expression values (NPX, Olink Proteomics arbitrary unit on log2 scale). All samples passed quality control in both panels. Two proteins, IL-2RB and IL2, had a missing frequency of 100% and were removed from the analysis.

#### Stool sample collection and fecal calprotectin measurement

Participants were asked to collect stool samples at home the day before a study visit. If this was deemed unfeasible, collecting the sample a day before or after the subsequent bowel movement was allowed. After completion of the procedure that was explained and fully detailed in the sampling kit, samples were immediately stored in participants’ home freezers.^46^ Participants were asked to bring the sample fully frozen and as quickly as possible after collection (usually at the study visit the next day). Upon reception, the stool samples were immediately frozen at −80°C. Fecal calprotectin concentrations were determined as previously described using the fCAL ELISA Kit (Bühlmann) for the extraction and quantitative determination of human calprotectin in stool samples.^46^

#### Microbiota analysis by 16S rRNA sequencing

Fecal DNA extraction and microbiota and bacterial profiling were carried out as described previously.^47^^,^^48^ Deep sequencing was performed on a MiSeq platform (2 × 250 PE reads, Illumina). All samples were randomized, and negative controls (PCR and extraction controls) were taken along and sequenced. After demultiplexing with sdm as part of the LotuS pipeline (v. 1.60), without allowing for mismatches, fastq sequences were further analyzed per sample using DADA2 pipeline (v. 1.6) with SILVA taxonomy database (v 138.1).

#### Analysis of total SCFA concentrations in fecal samples

Fecal samples (100 mg) were suspended in 1 mL of saturated NaCl (36%) solution. An internal standard (50 μL of 10.7 μM 2-ethylbutyric acid in MQ water) was added and the samples were homogenized using glass beads. After addition of 150 μL H_2_SO_4_ 96%, SCFAs were extracted with 3 mL of ether. The ether layer was collected and dried with Na_2_SO_4_ (150 mg). The supernatant (0.5 μL) was analyzed using gas chromatography with flame ionization detection (Agilent, Santa Clara, California, USA). The system was equipped with a DB FFAP analytical column (30 m × 0.53 mm ID, 1.0 μm; Agilent) and helium GC grade (5.6) was used as carrier gas with a constant flow of 4.2 mL/min. The initial oven temperature was held at 100°C for 3 min, ramped with 4°C/min to 140°C (isothermal for 5 min) and further with 40°C/min to 235°C (isothermal for 15 min). The resulting chromatograms were processed using ChemStation (Agilent Technologies).

#### Lactulose mannitol ratio testing

Urine was collected after an overnight fast and no intake other than water was allowed until the end of the experiment. This *in vivo* permeability test, performed after an overnight fast, was a standard differential urinary sugar excretion test. Participants were asked to provide a baseline urine sample and empty the bladder. Then, within 5 min after emptying the bladder, participants were asked to drink the test solution within 1 min in the presence of the investigator. The test solution consisted of 5 g of lactulose (EG) and 2 g of D-mannitol (Sigma-Aldrich) dissolved into 150 mL of water.^49^ Both sugars are absorbed and renally eliminated after absorption. Urine was collected 2 h after ingestion of the test solution in containers with 750 mg of neomycin sulfate to avoid bacterial proliferation. The lactulose-mannitol ratio (LMR) in the urine collection is a measure for small intestinal permeability.

#### Quantification of urinary lactulose and mannitol using gas chromatography-mass spectrometry analysis (GC-MS)

An internal standard mixture (200 μL) containing inositol (600 mg/L) and turanose (150 mg/L) was added to 20–150 μL standard solution containing mannitol (600 mg/L to 6 g/L), lactulose (100 mg/L to 1 g/L) or to 10–400 μL urine. Samples were diluted with demineralized water to 1 mL and 125 μL of the diluted samples was dried overnight at 50°C in a vacuum concentrator (RVC 2–18, Christ, Osterode am Hard, Germany). The sugars were converted into oximes by addition of 25 μL oxime reagent (250 mg hydroxylamine in 10 mL pyridine) and incubation at 75°C for 30 min. The samples were cooled down at −20°C for 10 min and derivatized with 25 μL BSTFA +1% TMCS for 35 min at 75°C. We injected 0.5 μL splitless into the GC-MS (Trace 1300, DSQ II XL) with an injector temperature at 250°C. Chromatographic separation was achieved with an Rxi-5ms column (30 m × 0.25 mm internal diameter, 0.25 μm film thickness; Restek, Bellefonte, PA) and a constant helium flow of 1 mL/min. The initial oven temperature of 100°C was kept isothermal for 3 min, ramped to 210°C with 30°C/min, increased to 270°C with 15°C/min, subsequently to 290°C with 30°C and was held for 10 min. The Rxi-5ms column was conditioned at 310°C for 10 min. Mass spectrometric detection was performed by electron impact in full scan mode (2 scans/s). M/z 361 was used to determine the area under the curve for lactose, sucralose and turanose, m/z 319 and 318 were used for mannitol and inositol, respectively. Data was processed by Excalibur (Thermo scientific, Pittsburgh, PA). When values were obtained lower than the limit of quantification (e.g., 5 mg/L for lactulose), 2.5 mg/L was used for further analysis. To increase comparability to other studies, the ratio of the fractional extraction of lactulose (FEL) and fractional excretion of mannitol (FEM) was reported.

### Quantification and statistical analysis

Non-parametric Wilcoxon rank-sum test and Wilcoxon signed-rank test were used for independent and paired testing, respectively. Permutational Multivariate Analysis of Variance Using Distance Matrices (PERMANOVA) was used to address the dynamics of Olink serum proteomics (adonis2 function from R vegan package 2.6–4). Principal components analysis of visualization of Olink serum proteomics was performed with olink_pca_plot function from R package OlinkAnalyze 3.4.1. The olink_lmer function (R package OlinkAnalyze) was used to perform linear mixed model per protein using weight and Treatment (baseline, high-AGEs or low-AGEs) as fixed variables and subject and sequence of the intervention as random variables. To address specific differences at protein level, a linear mixed model per protein was performed both in the inflammatory and cardiometabolic panels, using variables Treatment, Timepoint and Sequence as cofounders, and participant ID as a random variable. Microbiota alpha diversity was estimated using the function estimate_richness (R package -phyloseq 1.42), using Observed, Shannon, Simpson, and inverse Simpson measures. Principal coordinates analysis of the microbiota data was performed using Bray-Curtis’ dissimilarity index. The ASV abundance matrix was centered log-ratio (CLR)-transformed with ‘codaSeq.clr’ in the CoDaSeq (v.0.99.6), as previously described.^50^ Rarefaction false discovery rate (FDR) was used to adjust for multiple testing.^51^ Because of the exploratory character of the study, uncorrected *p*-values are given, except for the analyses regarding the microbiota and proteomics, given the extent of the datasets and multitude of testing. For all statistical testing, and N of twenty participants at three timepoints was used, unless otherwise specified. Depending on normality testing using Shapiro-Wilk, mean and standard deviation was used to define center and spread of the data, whereas median and interquartile ranges were used when data were not normally distributed. Further details can be found in legends of figures and tables as well as in the results section. No custom statistical computer coding was used in this paper. Any used code or additional information required to reanalyze the data reported in this work paper is available from the lead contact upon request. For analysis of the FFQ, the statistical program IBM SPSS Statistics 27 was used. For the other analyses, R statistical software version 4.2.2 was used.

### Additional resources

This study has been registered with ClinicalTrials.gov, NCT06547190.

## Supplemental information

Document S1. Figures S1–S7 and Tables S1–S4 and S7

Table S5. Macronutrient and CML intake

Table S6. Physical activity per participant per period

Data S1. Study protocol

Document S2. Article plus supplemental information
