# Supplementary material for: Cooking methods affect advanced glycation end products and lipid profiles: A randomized cross-over study in healthy subjects
Source: Cell Rep Med. 2025 Apr 24;6(5):102091. doi: 10.1016/j.xcrm.2025.102091 (PMC12147844; doi:10.1016/j.xcrm.2025.102091)
Supplement: Document S1. Figures S1–S7 and Tables S1–S4 and S7 [file mmc1.pdf]

Cell Reports Medicine, Volume *6*

Supplemental information

Cooking methods affect advanced glycation end products and lipid profiles: A randomized cross-over study in healthy subjects

Judith Wellens, Eva Vissers, Anaïs Dumoulin, Sien Hoekx, Julie Vanderstappen, Joke Verbeke, Roman Vangoitsenhoven, Muriel Derrien, Bram Verstockt, Marc Ferrante, Christophe Matthys, Jeroen Raes, Kristin Verbeke, Séverine Vermeire, and João Sabino

SUPPLEMENTARY TABLES

Meals	Low AGE CML content	High AGE CML content	Absolute difference
Codfish with green asparagus and cherry tomatoes	6,625	10,682	4,057
Mac and cheese with ham and brussels sprouts	18,103	26,079	7,976
Haddock with zucchini and potatoes	5,173	8,790	3,617
Turkey with carrots and potatoes	5,727	14,112	8,386
Wraps with chicken and vegetables	5,772	12,264	6,492
Noodles with chicken	5,060	12,206	7,145
Meat balls with tomato and potatoes	9,301	15,024	5,723
Codfish with leek and potatoes	6,352	7,209	858
Chicken and sweet potato	4,712	11,526	6,814
Salmon with dill and vegetables	8,988	12,691	3,703
Turkey with broccoli and potatoes	5,183	11,530	6,348
Wraps with chicken and tomato salsa	3,566	7,278	3,712
Spicy Thai curry with beef	4,626	23,513	18,887
Sweet rice with minced meat	6,709	11,699	4,990
Apple and pear crumble	6,112	8,922	2,809
Tomato and mozzarella snack	4,708	5,323	615
Bacon and eggs	3,185	27,667	24,482
Granola with yoghurt	571	863	292
Average	6,137	12,632	6,495

Table S1: Average CML content (kU CML) per recipe for the Low AGEs cooking methods and the High AGEs cooking methods respectively. Related to Figure 1.

	Baseline		High AGEs diet		Low AGEs diet	
	Average CML intake (kU CML/day)	Average CML intake per kcal (kU CML/day)	Average CML intake (kU CML/day)	Average CML intake per kcal (kU CML/day)	Average CML intake (kU CML/day)	Average CML intake per kcal (kU CML/day)
HL	17,267	11.69	21,304	11.17	10,672	5.79
LH	21,823	11.33	19,657	11.17	10,508	5.85
Average	19,545	11.51	20,480.5	11.17	10,590	5.82

Table S2: Calculated average CML intake and CML intake per kcal intake per period per group. One participant in the HL group was excluded from the analysis due to incomplete data. One participant in the LH group was excluded due to drop out, HL: N=10, LH: N=9. Related to Figure 2.

AGE	Baseline	High	Low	P-value B-H	P-value B-L	P-value H-L
All participants						
CML	83,470 (72,659 – 97,234)	61,724 (50,511 – 66,235)	48,297 (41,362 – 59,288)	**0.0003**	**0.01**	**0.001**
Pyrraline	2,124,826 (1,730,708 – 3,408,788)	1,568,514 (1,322,252 – 2,388,368)	1,382,254 (1,015,407 – 1,582,457)	>0.1	**0.008**	0.053
MG-H1	484,364 (383,488 – 626,522)	308,532 (180,059 – 407,423)	175,654 (119,010 – 233,620)	**0.0007**	**0.002**	**0.004**
High-to-Low AGEs group						
CML	92,141 (77,261 – 99,938)	64,582 (55,817 – 74,017)	53,324 (45,268 – 58,905)	**0.003**	**0.002**	**0.002**
Pyrraline	2,171,455 (1,759,606 – 3,198,771)	1,710,712 (1,343,841 – 2,398,187)	1,369,478 (849,504 – 1,788,911)	>0.1	>0.1	>0.1
MG-H1	578,624 (447,930 – 634,213)	352,254 (294,983 – 409,812)	203,039 (148,991 – 227,313)	**0.02**	**0.001**	**0.01**
Low-to-High AGEs group						
CML	78,077 (68,617 – 92,394)	57,708 (40,195 – 62,551)	44,074 (38,341 – 59,177)	**0.04**	**0.01**	>0.1
Pyrraline	1,930,914 (1,758,904 – 3,358,742)	1,397,636 (1,283,612 – 2,373,365)	1,430,123 (1,202,258 – 1,513,478)	>0.1	**0.004**	>0.1
MG-H1	387,549 (232,782 – 589,067)	183,900 (134,972 – 373,695)	138,737 (87,882 – 244,587)	**0.02**	**0.004**	0.098

Table S3: Measurement of advanced glycation end products in the serum using mass spectrometry. Median values and IQRs are reported. Paired Wilcoxon test. Analysis of N-(5-hydro-5-methyl-4-imidazolon-2-yl)-ornithine (MG-H1), N-(carboxymethyl)lysine (CML), and pyrraline. B-H: comparison Baseline to High-AGEs diet. B-L: Comparison Baseline to Low-AGEs diet. H-L: Comparison High – to Low-AGEs diet, N=20. Related to Figure

All participants					
Variable	Baseline	T1	T2	P-value B-T1	P-value T1-T2
Weight (kg)	65.8	65.1	64.9	**0.0005**	**0.03**
Calprotectin (µg/g)	30.0	30.0	30.0	> 0.1	> 0.1
CRP (mg/L)	1.1	0.6	0.6	**0.01**	> 0.1
Total cholesterol (mg/dL)	168	163	166	**0.007**	> 0.1
HDL-cholesterol (mg/dL)	70	68	63	**0.03**	> 0.1
LDL-cholesterol (mg/dL)	101	95	96	0.06	> 0.1
Triglycerides (mg/dL)	74	58	58	**0.001**	> 0.1
Fasting glucose (mg/dL)	83	84	84	> 0.1	0.07
Handgrip (kg)	31	33	33	**0.001**	> 0.1
High-to-Low AGEs group					
Weight (kg)	73.1	72.2	72.5	**0.002**	> 0.1
Calprotectin (µg/g)	30.0	30.0	30.0	> 0.1	> 0.1
CRP (mg/L)	1.3	0.8	1.1	> 0.1	> 0.1
Total cholesterol (mg/dL)	179	163	159	**0.01**	> 0.1
HDL-cholesterol (mg/dL)	65	61	59	**0.02**	> 0.1
LDL-cholesterol (mg/dL)	112	96	100	> 0.1	> 0.1
Triglycerides (mg/dL)	88	65	63	**0.001**	> 0.1
Fasting glucose (mg/dL)	82	84	80	> 0.1	0.07
Handgrip (kg)	34	43	44	**0.009**	> 0.1
Low-to-High AGEs group					
Weight (kg)	61.6	60.1	59.9	**0.03**	> 0.1
Calprotectin (µg/g)	30.0	30.0	30.0	> 0.1	> 0.1
CRP (mg/L)	0.8	0.6	0.6	0.058	> 0.1
Total cholesterol (mg/dL)	158	158	168	> 0.1	**0.04**
HDL-cholesterol (mg/dL)	75	73	80	> 0.1	0.05
LDL-cholesterol (mg/dL)	83	83	91	> 0.1	**0.02**
Triglycerides (mg/dL)	55	50	54	0.07	> 0.1
Fasting glucose (mg/dL)	83	81	84	> 0.1	> 0.1
Handgrip (kg)	27.7	32.3	30.7	0.07	> 0.1

Table S4: Anthropometric measurements and laboratory results at baseline, after the first interventional period (T1) and after the second interventional period (T2) for all participants (N=20), High-to-Low AGEs group (N=11) and Low-to-High AGEs group (N=9). Related to Figure

Questions	
Q1	How often do you steam food?
Q2	How often do you boil food in water?
Q3	How often do you prepare food in the microwave?
Q4	How often do you fry food in a pan?
Q5	How often do you stir-fry food?
Q6	How often do you grill food
Q7	How often do you prepare food in the oven?
Q8	How often do you toast bread?
Q9	How often do you fry food or eat fried foods?
Q10	How often do you prepare food yourself and then put it in the refrigerator to eat later?
Q11	How often do you eat home-cooked meals?
Q12	How often do you eat out, order food or eat readily prepared foods?
Answer	
A1	< 1 once a month
A2	1-3 times a month
A3	Once a week
A4	2-3 times a week
A5	4-6 times a week
A6	Daily

Table S7: Baseline questionnaire (questions and answer options) on use of cooking methods. Related to Figure 1.

Supplementary Figures

	High-dAGEs	Low-dAGEs
Day 1	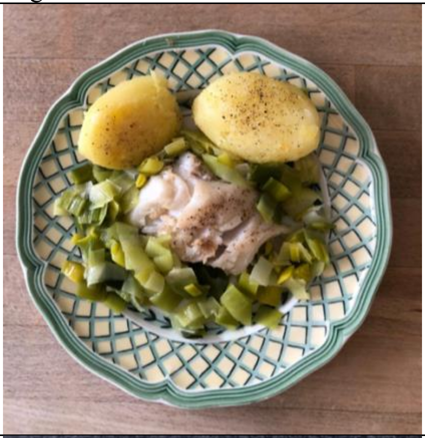	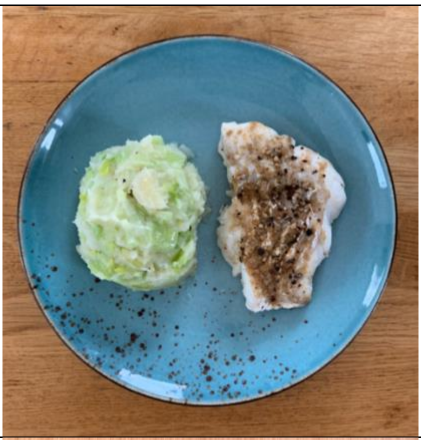
Day 2	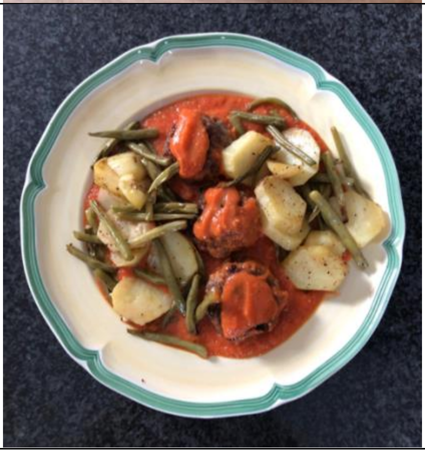	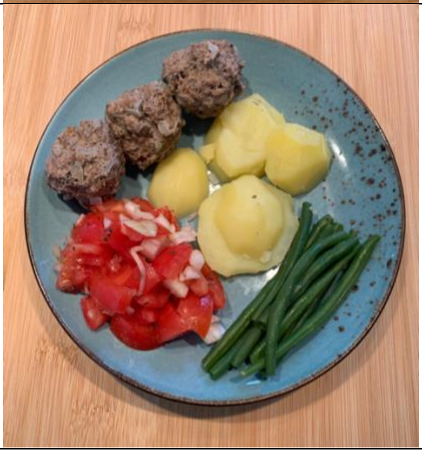

Figure S1: Examples of meals: Day 1 – Cod with leek and potatoes. Day 2 – minced meat balls with potatoes, onion, tomatoes, and green beans. Related to Figure 1A.

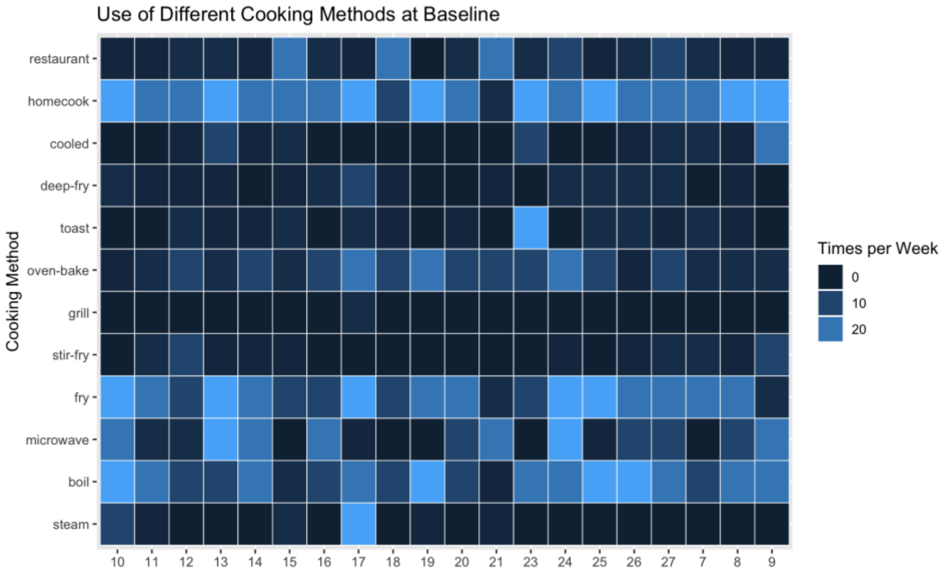

Figure S2: Baseline use of different cooking methods at baseline. Depiction of the frequency of use of different cooking methods by participant (x-axis, depicts one participant per column), N=20. Related to Figure 1A

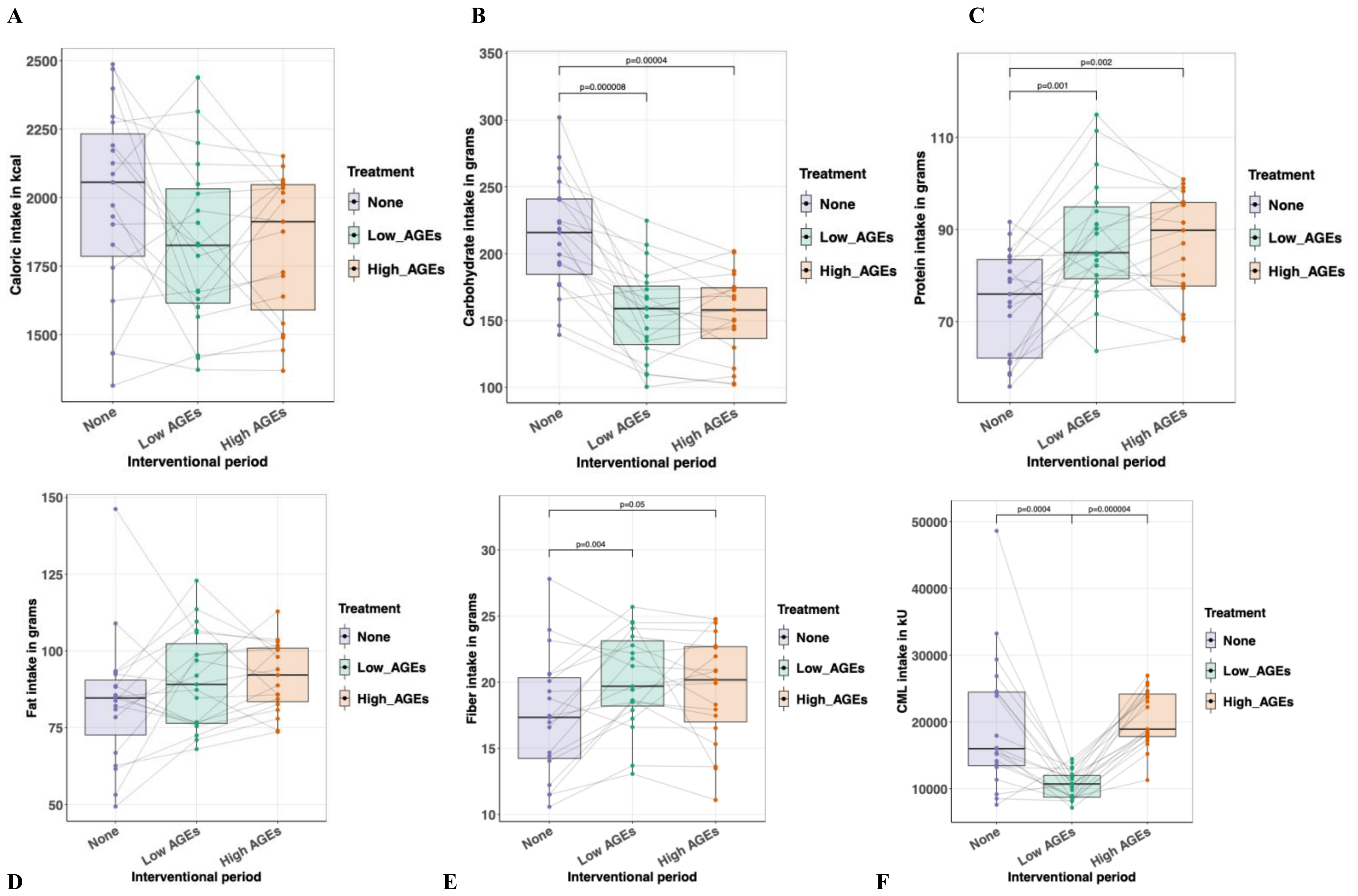

Figure S3: Energy, macronutrient and CML intake. Total energy (A), macronutrient (Carbohydrate B, Protein C, Fat D, Fiber E) and estimated CML intake (F) per dietary interventional period. Wilcoxon signed rank test. CML: carboxymethyl lysine. Related to Figure 2

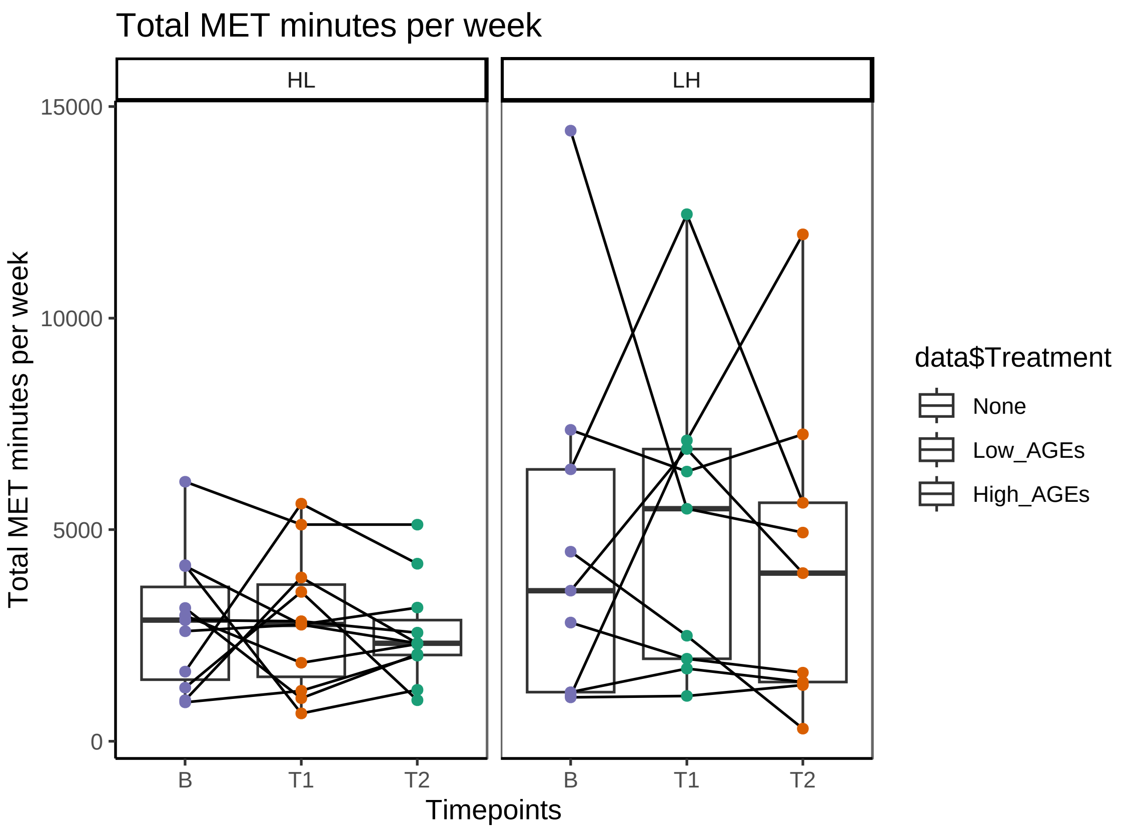

Figure S4: International Physical Activity Questionnaire. Total MET minutes per week per sequence. HL: high-to-low-AGEs), LH: low-to-high-AGEs, MET: metabolic equivalent. Wilcoxon signed rank test, p>0.1, N=20. Related to Figure 2.

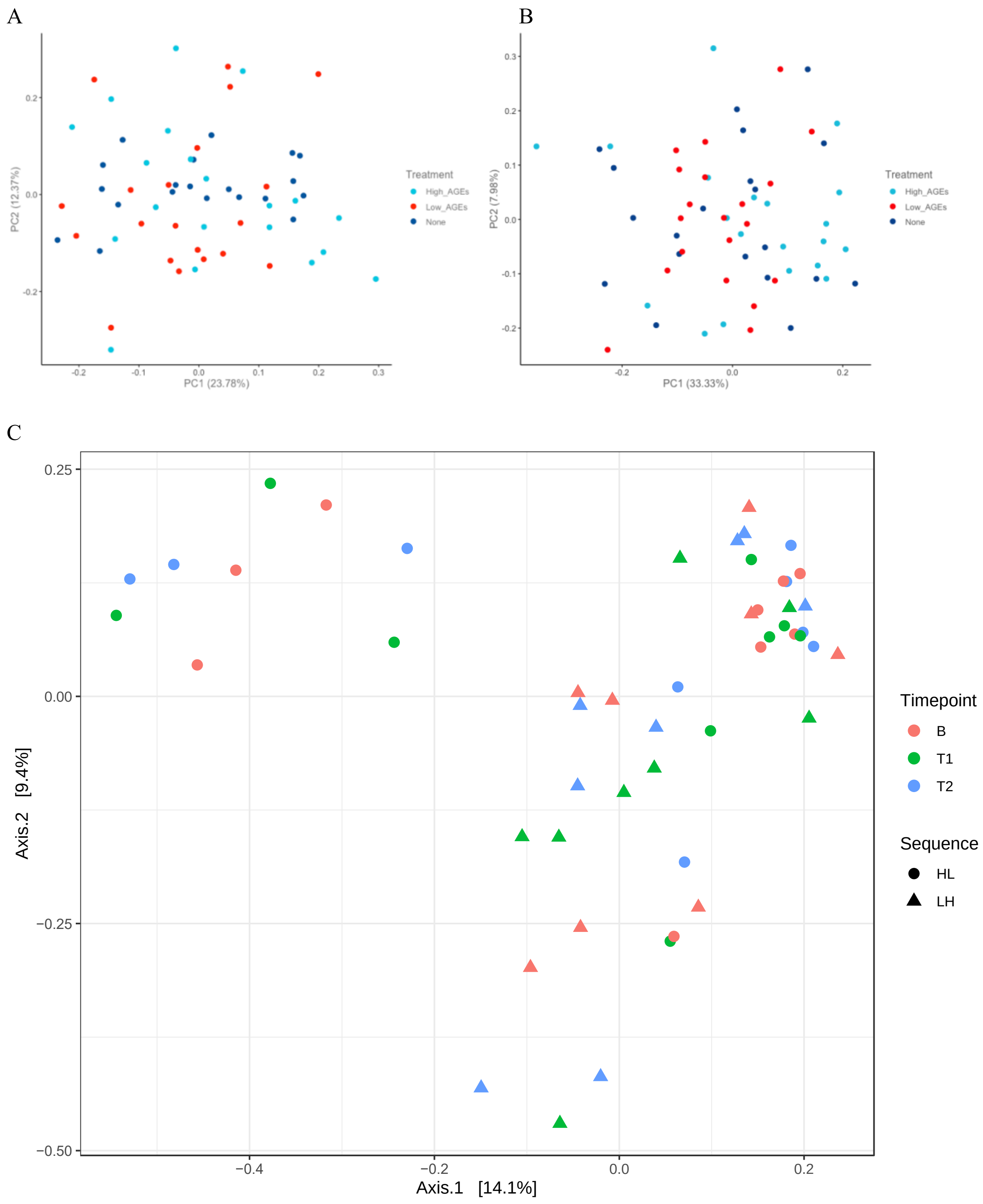

Figure S5: Serum proteomics Principal Components Analysis plot. Principal Components Analysis plot (PCoA) depicting changes in the serum proteomic profile according to dietary intervention for the inflammatory panel (panel A) and the cardiometabolic panel (panel B) and combined (panel C). B (baseline), T1 (timepoint 1), T2 (timepoint 2), HL (high-to-low-AGEs), LH (low-to-high-AGEs), N=20. Related to Figure 3.

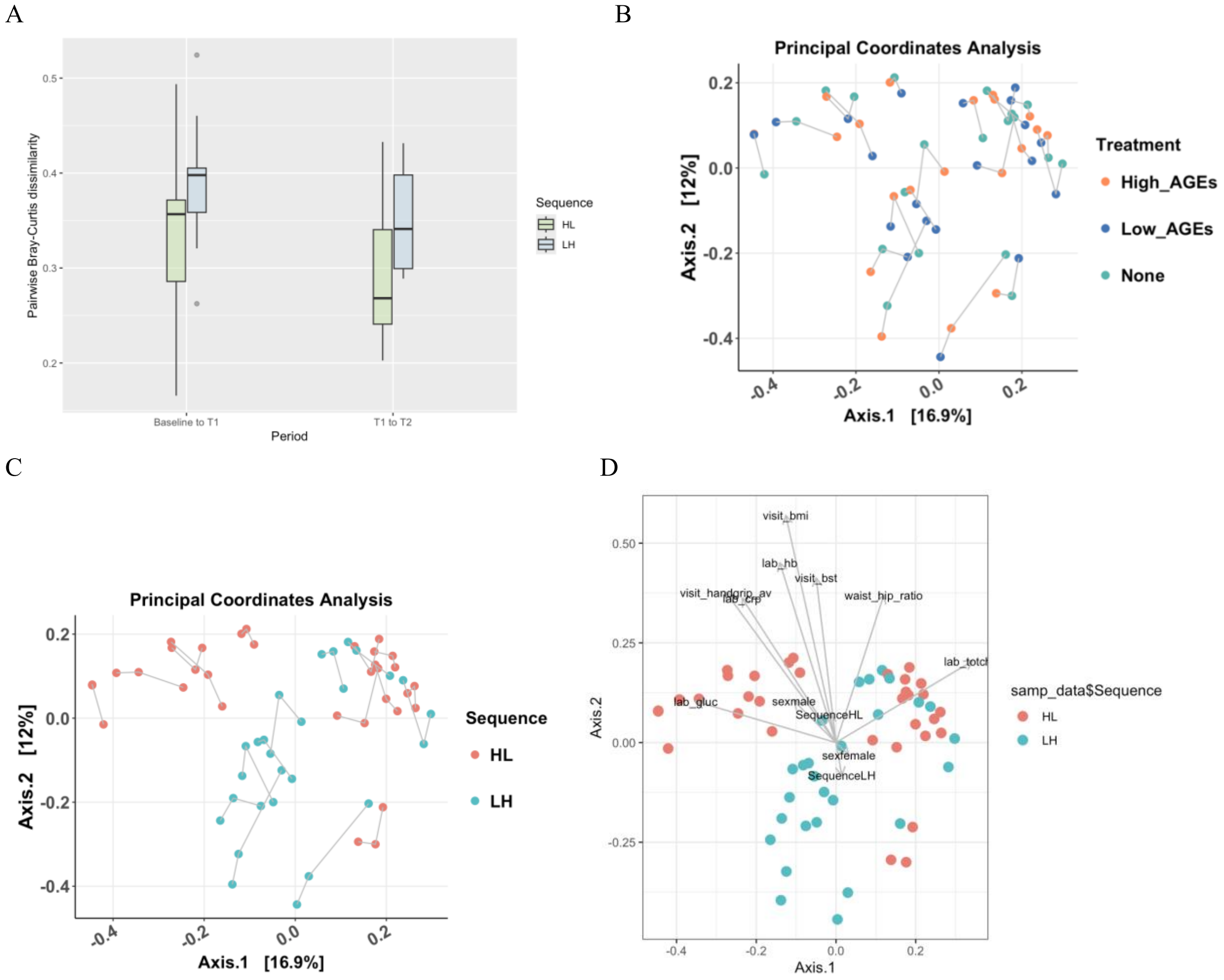

Figure S6: Gut microbiota. A: Pairwise Bray-Curtis dissimilarity at different timepoints per sequence. The gut microbial composition in the samples from the low-to-high AGEs group become more homogeneous (composition wise) after the second period. The Principal Coordinates Analysis (PCoA) shows no distinct clusters in the gut microbial composition based on Treatment (B) but does according to sequence (C): Low-to-High AGEs diet (LH) or High-to-low AGEs diet (HL)). D: PCoA showing that any of the observed variation is explained by BMI (p=0.001), handgrip strength (p=0.002), hemoglobin (p=0.004), CRP (p=0.004), Bristol Stool Score (p=0.006), waist-hip ratio (p=0.01), total cholesterol (0.01) and fasting glucose (p=0.01), but not a change serum AGEs, N=20. Related to Figure 3.

A

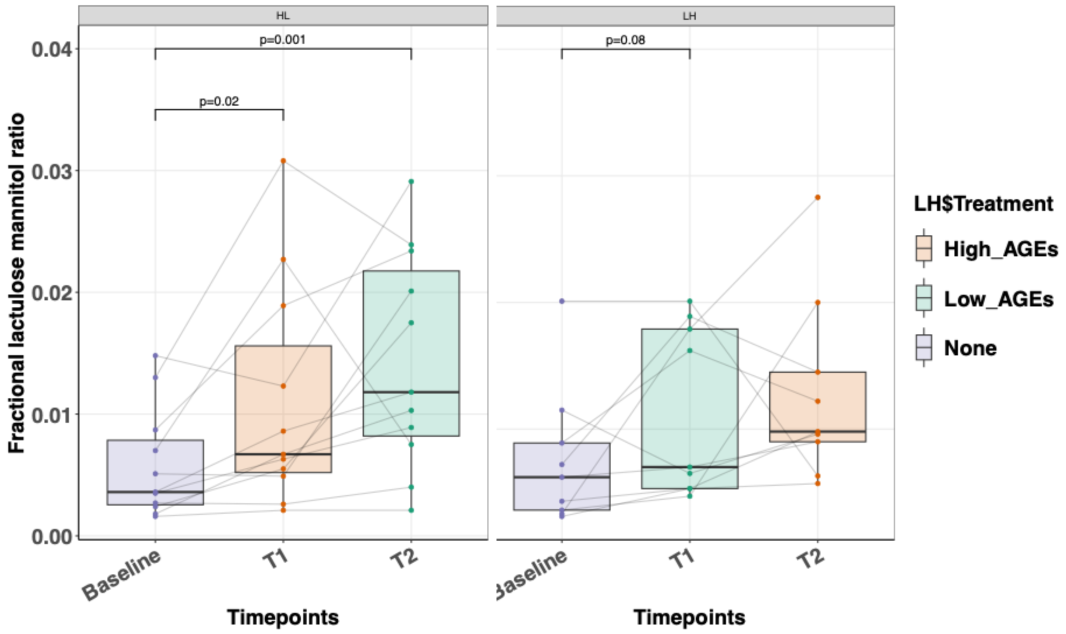

B

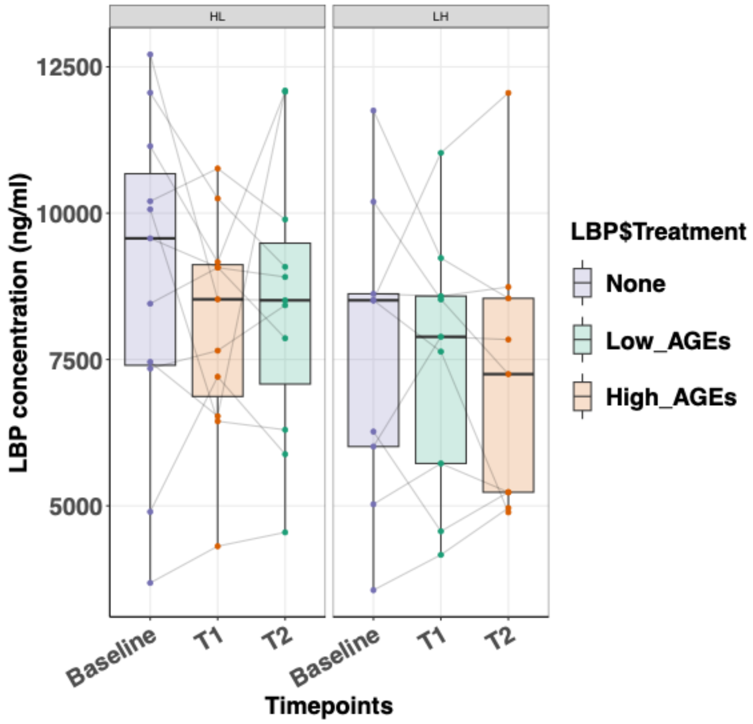

Figure S7: Intestinal permeability. Panel A: Results of the fractional lactulose-mannitol ratio test at every timepoint (baseline, after the first interventional period (T1) and after the second interventional period (T2) per sequence. We report an increased intestinal permeability in het LH group after consuming the low-AGEs diet (paired Wilcoxon signed rank test p=0.001). Panel B: Results of the LBP ELISA at every timepoint (baseline, after the first interventional period (T1) and after the second interventional period (T2) per sequence, N=20. Related to Figure 3.
