# Supplementary material for: Cooking methods affect advanced glycation end products and lipid profiles: A randomized cross-over study in healthy subjects
Source: Cell Rep Med. 2025 Apr 24;6(5):102091. doi: 10.1016/j.xcrm.2025.102091 (PMC12147844; doi:10.1016/j.xcrm.2025.102091)
Supplement: Data S1. Study protocol [file mmc4.pdf]

**Protocol Full Title**: Study on Treating by Eating: role of Advanced glycation end products (AGEs) on Mucosal barrier and Microbiome

## Protocol Acronym/short title: STEAMM

STEAMM
Study on Treating by Eating: role of AGEs on Mucosal barrier and Microbiome

## Version and date of final protocol: version 3

## Sponsor:

Name: KULeuven
Address: Herestraat 49, 3000 Leuven

## Principal Investigator:

Name: Prof. João Sabino MD PhD
Address: Herestraat 49, 3000 Leuven
Telephone: 016 341770
Fax: 016 344419
Email: joao.sabino@uzleuven.be

## Sub-investigator:

Name: Séverine Vermeire
Address: Herestraat 49, 3000 Leuven
Telephone: 016 342360
Fax: 016 344419
Email: severine.vermeire@kuleuven.be

Name: Jeroen Raes
Address: Herestraat 49, 3000 Leuven
Email: jeroen.raes@kuleuven.be

## Signatures

____________________________ ______________________

Principal Investigator Date

Print Name:

____________________________ ______________________

Date

Print Name:

## Table of Contents

1. Study Synopsis ........ 6
2. Background and rationale ........ 8
3. Trial objectives and Design ........ 9
   3.1 Trial objectives ........ 9
   3.2 Exploratory endpoints ........ 9
   3.3 Trial Design ........ 10
   3.4 Study diagram ........ 11
   3.5 Trial Flowchart ........ 11
4. Selection and withdrawal of subjects ........ 12
   4.1 Inclusion criteria ........ 12
   4.2 Exclusion criteria ........ 12
   4.3 Expected duration of trial ........ 13
5. Trial Procedures ........ 13
   5.1 By visit ........ 13
   5.2 Definitions ........ 14
   5.3 Laboratory tests ........ 15
   5.4 Other investigations ........ 15
6. Assessment of Safety ........ 16
   6.1 Specification, timing and recording of safety parameters ........ 16
   6.2 Procedures for recording and reporting adverse events (AE) ........ 16
   6.3 Treatment stopping rules ........ 17
7. Statistics ........ 17
   7.1 Sample size ........ 17
   7.2 Analysis ........ 17
8. Direct access to source data and documents ........ 18
9. Ethics and regulatory approvals ........ 18
10. Data Handling ........ 19
11. Data Management ........ 19
12. Translational research ........ 20

13. Publication Policy ........ 20
14. Insurance/Indemnity ........ 20
15. Financial Aspects ........ 20
16. References ........ 21
Appendices ........ 22
Appendix 1: Questionnaire ........ 22
Appendix 2: Menu ........ 24
Appendix 3: Recruitment flyer ........ 30
Appendix 4: Frequently asked questions ........ 31

# 1. Study Synopsis

| | |
|---|---|
| Title of clinical trial | Study on Treating by Eating: role of advanced glycation end products (AGEs) on mucosal barrier and microbiome |
| Protocol Short Title/Acronym | STEAMM |
| Sponsor name | KU Leuven |
| Principal Investigator | Prof. João Sabino MD PhD |
| Medical condition or disease under investigation | Inflammatory bowel diseases |
| Purpose of clinical trial | To assess the effect of steam-cooking vs. pan-frying and grilling on intestinal inflammation, permeability and gut microbiome. |
| Primary objective | (1) To assess the influence of different cooking methods/AGEs on (intestinal) inflammation. |
| Secondary objective(s) | (1) To assess the influence of different cooking methods on intestinal permeability.<br><br>(2) To assess the influence of different cooking methods on the microbiota and its function. |

| | |
|---|---|
| | (3) Assess the influence of AGEs intake on the intestinal inflammation, permeability, and microbiota. |
| Trial Design | Open-label, interventional, cross-over pilot study |
| Exploratory Endpoints | (1) Change in fecal calprotectin after each arm of the diet.<br>(2) Change in serum C-reactive protein (CRP) after each arm of the diet.<br>(3) Change in serum OLINK proteomics after each arm of the diet.<br>(4) Change in intestinal permeability measured with lactulose mannitol urine test after each arm of the diet.<br>(5) Gut microbiome alterations evaluated with 16S rRNA sequencing<br>(6) Short chain fatty acids (SCFA) dynamics during the trial.<br>(7) Faecal metabolomics dynamics during the trial.<br>(8) Change in lipopolysaccharide (LPS) and flagellin levels after each arm of the diet.<br>(9) Influence of genetics in the dynamics of faecal calprotectin after each arm of the diet. |
| Sample Size | N = 20 healthy volunteers |
| Summary of eligibility criteria | - Healthy individuals aged 18 or older;<br>- No current chronic illness;<br>- Normal BMI (18,5-25 kg/m$^2$);<br>- Absence of any eating disorder, irritable bowel syndrome (IBS), inflammatory bowel disease (IBD) or a family history of IBD;<br>- No antibiotic, prebiotic or probiotic use in the past 6 months;<br>- No use of aspirin or NSAIDs 4 weeks before enrollment; |

| | |
|---|---|
| | - No current smoking;<br>- Women that are pregnant, nursing, or planning pregnancy will be excluded;<br>- Availability of a well-equipped kitchen and freezer space.<br>- No food allergies or intolerances to any components of the study diet |
| Maximum duration of treatment of a Subject | 4 weeks |
| Version and date of final protocol | Version 3 15/12/2021 |
| Version and date of protocol amendments | NA |

## 2. Background and rationale

Traditionally, the value of food has been measured by its ability to provide nutrients to the host.(1) Although this remains important, research interest is redirecting towards production methods and food additive use because of the apparent coinciding of ultra-processed food consumption in middle- to high income countries with the Western epidemic in chronic diseases such as the metabolic syndrome and cancer.(2–4) Although food processing has several advantages like prolonging shelf-life, increasing palatability and decreasing costs to the consumer, there are possible downsides to be considered.(4) Thermal heating alters the chemical composition of food through an intricate process called 'the Maillard reaction'. This occurs when reducing sugars react with free amino groups of amino acids or proteins, giving rise to the Maillard reaction products, including advanced glycation end products (AGEs).(5,6) Interestingly, AGEs have been associated in both human and animal studies with a wide range of diseases, such as type 2 diabetes, cardiovascular, and renal diseases.(7–11) The pathogenic effects of AGEs is thought to be related to their capacity to generate oxidative stress and inflammation by binding to cell-surface receptors or cross-linking with body proteins and thereby altering their function.(12) Furthermore, although a small proportion is absorbed in the small intestine, the majority of dietary AGEs will escape absorption and become substrates for the microbiota. This means that AGEs are capable of modifying the host gut microbiome to a more deleterious composition, as has previously been measured in faeces in rodents, healthy volunteers, and patients with ulcerative colitis(5,13,14). Furthermore, AGEs may have an additional local effect

on the gastro-intestinal tract. This is illustrated by data on sustained and worsened intestinal inflammation through elevated expression of AGE receptors and NF-KB in the gut in both susceptible rodents and patients with inflammatory bowel disease (IBD) when challenged with diets high in AGEs.(7,15–19)

The production of dietary AGEs occurs upon heating of food both in industrial food processing and home-cooked meals. The amount produced, increases with an increased cooking time, temperature, the absence of moisture and a more alkaline environment, so that for example frying and grilling will produce more AGEs than boiling and steaming of foods.(13,20) Moreover, foods high in fat and protein typically contain higher levels of AGEs than carbohydrate-rich foods.(13,20) This means that the amount of AGEs people consume can be altered by changing their diet, suggesting that dietary modifications might be considered as a novel therapeutic target.(12)

Therefore, we propose a pilot study in healthy individuals to compare cooking methods that are known to produce AGEs to a different extent, and assess their effects on intestinal inflammation, intestinal permeability, and the gut microbiome.

# 3. Trial objectives and Design

## 3.1 Trial objectives

(1) We hypothesize that a diet high in AGEs has a pro-inflammatory potential when compared to a diet low in AGEs.
(2) We hypothesize that a diet high in AGEs increases intestinal permeability.
(3) We hypothesize that a diet high in AGEs generates a different gut ecology and that this will be reflected in measurable population differences and functionality of the microbiome.
(4) We hypothesize that if a diet high in AGEs plays a role in maintaining and worsening inflammation, a diet low in AGEs could be considered as a novel therapeutic strategy in inflammatory bowel diseases. As such we want to determine the feasibility of such dietary intervention.

## 3.2 Exploratory endpoints

(1) Change in faecal calprotectin after each arm of the diet.
(2) Change in serum C-reactive protein (CRP) after each arm of the diet.
(3) Change in serum OLINK proteomics after each arm of the diet.
(4) Change in intestinal permeability measured with lactulose mannitol urine test after each arm of the diet.
(5) Gut microbiome alterations evaluated with 16S rRNA sequencing
(6) Short chain fatty acids (SCFA) dynamics during the trial.
(7) Faecal metabolomics dynamics during the trial.
(8) Change in lipopolysaccharide (LPS) and flagellin levels after each arm of the diet.
(9) Influence of genetics in the dynamics of faecal calprotectin after each arm of the diet
(10) Measure differences in AGEs produced in the cooked foods by mass spectrometry.

### 3.3 Trial Design

This is a 5-week open-label cross-over pilot study in healthy volunteers to compare the effects of a diet high in AGEs to a diet low in AGEs on intestinal inflammation and the gut microbiota.

We will recruit 20 healthy volunteers. Recruitment flyers (Appendix 3) will be dispersed at KULeuven. The subjects will be randomised to 2 groups of 10 subjects each: high-to-low group (HL group) and low-to-high group (LH group). To avoid confounding factors as much as possible, only one subject per household will be recruited in the study.

After randomization, both groups will be asked to keep a nutrition diary through the application FatSecret for the duration of the study. This application is a free to download application compatible with Apple and Android devices. Then, the HL group will start with a diet high in AGEs obtained through pan-frying or grilling of ingredients. The LH group will start with a diet low in AGEs obtained through steam-cooking or boiling food. After 2 weeks, the groups will switch interventions so that the HL group will adhere to the diet low in AGEs and the LH group to the diet high in AGEs (see figure).

The study participants will be provided with a list of recipes and cooking instructions for all meals and snacks for the whole duration of the trial. The menu and recipes were designed with the help of dieticians to assure a correct nutritional balance. All ingredients will be provided by the study team to every participant free of charge. A steam cooker will also be provided by the study team.

Blood, stool, and urine samples will be collected before and after a nutritional intervention, specifically at timepoints= 1 week, t= 3 weeks, and t=5 weeks. The questionnaire (Appendix 1) as well as the International Physical Activity Questionnaire (IPAQ), and a food frequency questionnaire will be taken at baseline, and at each visit. These data will be stored in a Redcap database. Fasting blood samples (up to 20 ml in SST tubes) will be taken at the predefined timepoints for evaluation of systemic inflammation. Lactulose mannitol testing will be performed for assessment of intestinal permeability. Stool samples will be collected for faecal calprotectin and intestinal microbiota analysis using in house collection kits. Smoking or drinking alcohol is not allowed during the study period. The subjects will be asked not to take any kind of medication unless approved by the research team. The patients will be asked to keep a nutrition diary (including beverages) through the smartphone application FatSecret to assess adherence to the protocol during intermediary visits and for further analysis.

All costs of the study will be covered by study budget. Subjects will not receive a financial compensation for participation in this study. When all samples are received and nutrition diaries are adequately filled in (documentation of 80% of meals or more), participants are rewarded with their steam cooker. All foods are provided by the study. A list of additional permitted (and prohibited) foods will be available for each participant, as well as a sheet with frequently asked questions (Appendix 2 and 4). When additional questions would arise, they can be asked during planned

contact moments. Additional questions can be asked by emailing or calling the study team. Contact information will be provided.

### 3.4 Study diagram

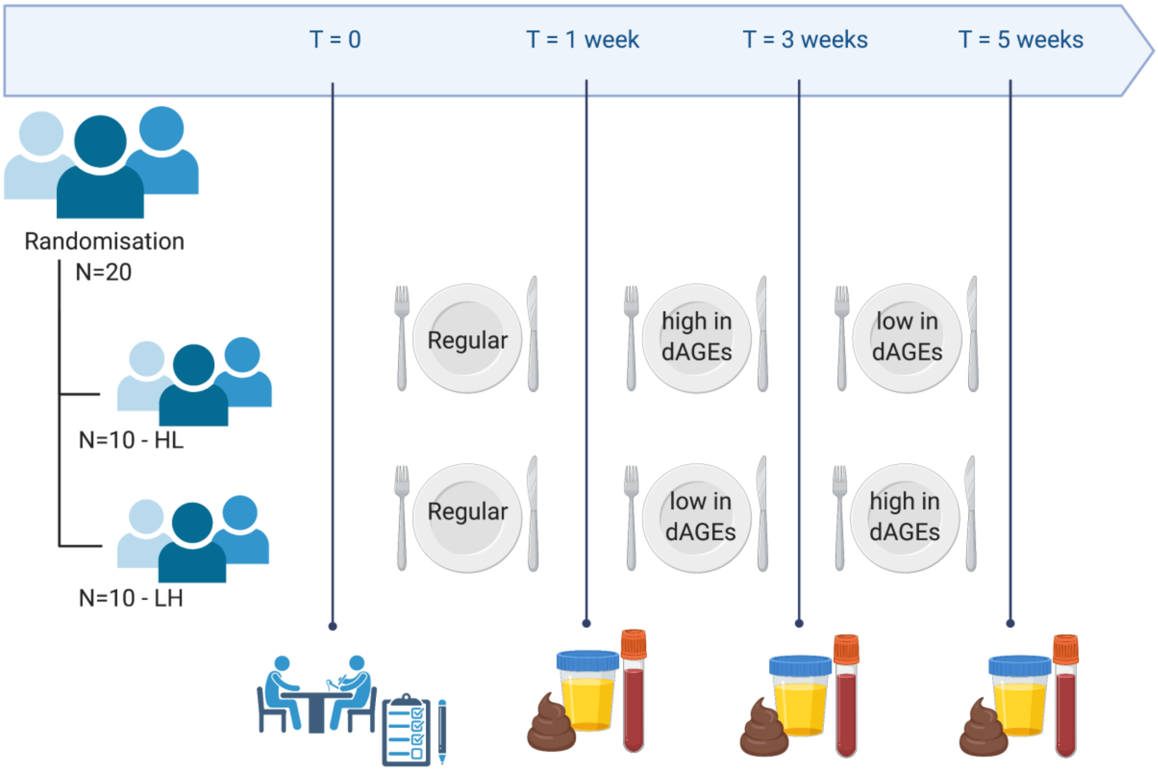


### 3.5 Trial Flowchart

| | Screening visit | T = 0 | T = 1 week | T = 3 weeks | T = 5 weeks |
|---|---|---|---|---|---|
| Informed consent | X | | | | |
| Verify inclusion and exclusion criteria and explain the study | X | | | | |

| | | | | | |
|---|---|---|---|---|---|
| History taking and physical examination<br><br>(Redcap) | | X | X | X | X |
| Questionnaire in Appendix 1, IPAQ and food frequency questionnaire<br><br>(Redcap) | | X | X | X | X |
| Food diary with FatSecret | X | X | X | X | X |
| Blood sample | | | X | X | X |
| Faecal sample | | | X | X | X |
| Lactulose mannitol test | | | X | X | X |

# 4. Selection and withdrawal of subjects

## 4.1 Inclusion criteria

In order to be eligible to participate in this study, an individual must meet all of the following criteria:

- Provide signed and dated informed consent form.
- Willing to comply with all study procedures and be available for the duration of the study.
- Aged 18 to 65 years old. Women of child-bearing age will be included if suitable methods of contraception are being used.
- Generally healthy, no current chronic illness. Prior illnesses and/or surgery are allowed, except if antibiotics have been used in the last 3 months.
- Normal body mass index (BMI), meaning between 18.5 – 25 kg/m$^2$.
- Absence of eating disorder (even when treated).
- Availability of refrigerator and freezer space.
- Availability of a well-equipped kitchen with oven, stove, pots and skillets, kitchen knives, a colander.

## 4.2 Exclusion criteria

Subjects will be excluded when:

- Unable to provide an informed consent

- Pregnancy, lactation or unsuitable methods of contraception
- Presence of a chronic disease, or any known conditions resulting in immunosuppression
- Past medical history of any eating disorder, irritable bowel syndrome (IBS), celiac disease, IBD or any other chronic intestinal disorder
- Prior bowel resection surgery other than appendectomy
- Family history of IBD
- Current smoking
- Current use of NSAIDs, aspirin, laxatives, anti-diarrheal medication, anticholinergic medications, narcotics, antacids, PPIs, or dietary supplements that cannot be stopped 4 weeks before the start of the trial
- Antibiotic, prebiotic or probiotic use in the past 6 months
- Presence of food allergies or intolerance to the components of the study diets
- Following a vegan or vegetarian diet
- Experienced diarrhoea withing the two weeks prior to screening

### 4.3 Expected duration of trial

The expected duration of the trial for participants will be 7 weeks. This includes 5 weeks for completing the study protocol and 2 weeks before the trial to plan the screening visits.

## 5. Trial Procedures

### 5.1 By visit

Screening visit

- Review medical history to determine eligibility based on inclusion/exclusion criteria.
- Explain the study protocol and informed consent form.
- Informed consent must be obtained prior to the subject entering the study, and before any protocol-directed procedures are performed.
- A unique subject identification number (subject number) will be assigned to each subject at the time of screening; this subject number will be used throughout the study.

T=0

- History taking and physical examination
- Obtain questionnaire with demographic data, medical history, current medication, last antibiotic use, smoking status. (cfr. Appendix 1)
- Review the details of the protocol.
- Review the nutrition diary (FatSecret) and stress its importance.
- Provide with stool sample collection kit.

T= 1, 3 and 5 weeks

- History taking and physical examination (Redcap).
- IPAQ questionnaire
- Food frequency questionnaire
- Questionnaire in Appendix 1 (follow-up)
- Hand grip
- Assess adherence using the application FatSecret.
- Collection of blood, urine, and stool samples.
- Review the nutrition diary and stress its importance.
- Review the details of the next diet.
- Provide with stool collection kit for the next week.

## 5.2 Definitions

**Demographics, Medical History, and Medication History Procedure**

Demographic information to be obtained will include year of birth or age, sex, ethnic origin as described by the subject.

Medical history to be obtained will include determining whether the subject has any significant conditions or diseases relevant to the study.

Medication history information to be obtained also includes any medication relevant to eligibility criteria stopped at or within 3 months prior to signing of informed consent.

**Physical Examination Procedure**

A baseline physical examination will consist of the following body systems: (1) eyes; (2) ears, nose, throat; (3) cardiovascular system; (4) respiratory system; (5) gastrointestinal system; (6) dermatologic system; (7) extremities; (8) musculoskeletal system; (9) nervous system; (10) lymph nodes; and (11) other. All subsequent physical examinations should assess clinically significant changes from the assessment prior baseline physical examination.

**Weight, Height**

A subject should have weight and height measured while wearing indoor clothing and without shoes. The standard for collecting height is centimetres without decimal places and for weight it is kilograms (kg) with 1 decimal place.

**Vital Sign Procedure**

Vital signs will include blood pressure and pulse (bpm).

**Waist circumference**

Waist circumference will be measured (cm).

**Hand grip**

Handgrip will be measured with hand dynamometer. This will be performed 3 times in the dominant hand.

**Documentation of Concomitant Medications**

Concomitant medication is any drug given during the study. These may be prescribed by a physician or obtained by the subject over the counter. All medication including vitamin supplements, over-the-counter medications, and oral herbal preparations, must also be recorded in the CRF.

## 5.3 Laboratory tests

### *Laboratory test:*

- Blood laboratory tests: Hemoglobin, white blood cell (WBC) count, WBC differentiation, thrombocytes, urea, creatinine, sodium, potassium, chloride, phosphate, calcium, magnesium, alkaline phosphatase, gamma-glutamyl transferase, alanine aminotransferase, aspartate aminotransferase, bilirubin, total cholesterol, HDL-cholesterol, LDL- cholesterol, triglycerides, fasting glucose, uric acid, C-reactive protein.
- OLINK serum proteomics: Serum samples will be used for proteomics studies using the OLINK platform. The OLINK inflammation panel allows the analysis of 92 biomarkers linked to inflammation processes.
- Genetic profiling: The genetic profile of all included participants will be characterized using a custom-made assay (molecular inversion probes – MIP- genotyping assay) covering all 242 known loci, and a genetic risk score for all loci combined will be calculated.
- Urine: lactulose/mannitol test (2-hour protocol).
- Stool samples: 16S rRNA sequencing, shotgun sequencing, SCFA measurement, metabolomics, measurement of LPS, flagellin levels, faecal elastase, estimation of the water content and measurement of the bacterial load (by flow cytometry). 16SrRNA sequencing, and measurements of water content and bacterial load will be performed at the VIB by the team of Jeroen Raes.

### *Storing and handling of specimens:*

- Blood specimens: Blood specimens up to 50 ml will be taken by the study team (3x SST, 1x EDTA, 1x fluoride). Only the blood collection at enrolment needs take place after an overnight fasting period.  All the samples will be aliquoted and stored at -80°C.
- Urine specimens: Intestinal permeability will be assessed through lactulose/mannitol urinary testing. Subjects will be asked to for a fasting urine sample after 8 hours of fasting, only water is permitted. 2 hours after drinking a solution with lactulose and mannitol, a second urine sample will be asked. After analysis, the urine will be stored at -80°C.
- Faecal specimens: Faecal samples will be aliquoted for measurement of the faecal calprotectin and intestinal microbiota. Aliquots will be stored at -80°C.

## 5.4 Other investigations

Only laboratory test will be performed.

### 5.5 Assessment of adverse events

Any adverse or serious adverse events that occurred between two visits should be recorded and the severity as well as the correlation to the investigational diet should be clarified.

## 6. Assessment of Safety

### 6.1 Specification, timing and recording of safety parameters

At each visit, a medical history will be taken, and a targeted physical examination performed (if necessary) to ascertain if there are any adverse events. Also, at every timepoint there will be blood taken, we will check if there are any alterations to baseline that might have a connection to the study protocol. The blood tests to be ran will include: Hemoglobin, WBC count, WBC differentiation, thrombocytes, urea, creatinine, sodium, potassium, chloride, phosphate, calcium, magnesium, alkaline phosphatase, gamma-glutamyl transferase, alanine aminotransferase, aspartate aminotransferase, bilirubin, total cholesterol, HDL-cholesterol, LDL-cholesterol and triglycerides, fasting glucose, uric acid, C-reactive protein.

### 6.2 Procedures for recording and reporting adverse events (AE)

Definitions of adverse events:

***Adverse Event (AE):*** Any untoward medical or psychological occurrence in a patient or necessarily have a causal relationship with this intervention. An adverse event can therefore be any unfavorable and unintended sign, symptom, or disease in any subject in a trial (including those in an untreated control group), whether considered related to the investigational psychological therapy / intervention or not.

***Serious Adverse Event (SAE) or Serious Adverse Reaction (SAR):*** any untoward medical or psychological occurrence that:

- Results in death,
- Is life-threatening
- Requires inpatient hospitalization or prolongation of existing hospitalization,
- Results in persistent or significant disability/incapacity

When an any of the above, mentioned AEs takes place it will be documented clearly in the patient medical record:

- Whether the event has been observed by the PI (or delegate) or reported by the subject.
- The date - and if possible, the time - of the onset of the reaction.
- If completely resolved, the duration of the reaction.
- The severity of the reaction (not to be confused with seriousness – see definitions).
- Any action taken regarding the psychological therapy / intervention.
- Any treatment/ medication given for the reaction, including dates.

- The outcome of the episode.

AEs that result in the subject withdrawing – or being withdrawn - from the study must be recorded for inclusion in the annual progress report to the Ethics Committee. If an SAE of SAR is suspected, the PI will be notified immediately (within 24 hours) and the EC will be notified. The study will be suspended until further notice.

### 6.3 Treatment stopping rules

Subjects may withdraw voluntarily from the study or the investigator may terminate a subject's participation. Subjects are free to withdraw from participation in the study at any time upon request. Patients should contact the investigator or other member of the research team to request withdrawal from the study, at which point data and specimens pertaining to the patient will be deleted/destroyed. Data and specimens from subjects who withdraw after collection may be analyzed.

An investigator may terminate a study subject's participation in the study if the study protocol has not been appropriately followed or if an adverse reaction occurs. If an SAE of SAR is suspected, the PI will be notified immediately (within 24 hours) and the EC will be notified. The study will be suspended until further notice.

## 7. Statistics

### 7.1 Sample size

We aim for a sample size of 20 subjects in total. Since this is a pilot study, a formal sample size calculation is not possible. The results of this study will enable estimating an effect size for a possible subsequent study on IBD patients.

### 7.2 Analysis

All analysis will be done after the end of the study. Interim analyses will be focused on the Hemoglobin, WBC count, WBC differentiation, thrombocytes, urea, creatinine, sodium, potassium, chloride, phosphate, calcium, magnesium, alkaline phosphatase, gamma-glutamyl transferase, alanine aminotransferase, aspartate aminotransferase, bilirubin, total cholesterol, HDL-cholesterol, LDL-cholesterol and triglycerides, fasting glucose, uric acid, C-reactive protein for safety considerations only.

Subjects will be asked to stop the trial when the protocol is not followed. Subjects with missing data will be excluded for the analysis that involves the missing data (e.g.: when no lactulose mannitol test was performed, the subject will be excluded for intestinal permeability testing, but not for faecal calprotectin if that value is available).

Descriptive statistics will be used to characterize the included cohort. Continuous data without normal distribution will be presented as median and interquartile range. Continuous data with normal distribution will be presented as average and standard deviation. Categorical data will be presented as absolute number and percentage.

The dynamics of several variables will also be compared over time as paired data (e.g. faecal calprotectin), using appropriate statistical tests (such as the Wilcoxon signed-rank test and the McNemar's test (2x2, paired data).

Given the multitude of data that will be collected, care shall be taken to account for multiple testing (e.g. use of false discovery rate in microbiome data analysis) before conclusions are drawn.

Of course, this is a pilot study and possible results will be evaluated and communicated as such. Possible significant statistical findings should (and will) be checked in a subsequent (and sufficiently powered) follow-up study.

## 8. Direct access to source data and documents

The investigator and the institution will permit trial-related monitoring, audits, EC review, and regulatory inspections (where appropriate) by providing direct access to source data and other documents (e.g. patients' case sheets, blood test reports, urine test and faecal material).

## 9. Ethics and regulatory approvals

The trial will be conducted in compliance with the principles of the Declaration of Helsinki (64th WMA General Assembly, Fortaleza, Brazil, October 2013), the principles of GCP and in accordance with all applicable regulatory requirements. This protocol and related documents will be submitted for review to Ethics Committee

If protocol amendments are desired, these will be submitted to the EC for approval. A copy of the Final Study Report will be submitted to the Ethics Committee after terminating the study.

The Study can and will be conducted only on the basis of prior informed consent by the Subjects, or their legal representatives, to participate in the Study. The Participating Site shall obtain a signed informed consent form (ICF) for all patients prior to their enrollment and participation in the Study in compliance with all applicable laws, regulations and the approval of the (local) Ethics Committee, if required. The Participating Site shall retain such ICFs in accordance with the requirements of all applicable regulatory agencies and laws.

The Investigator and the Participating Site shall treat all information and data relating to the Study disclosed to Participating Site and/or Investigator in this Study as confidential and shall not disclose such information to any third parties or use such information for any purpose other than the performance of the Study. The collection, processing and disclosure of personal data, such as patient health and medical information is subject to compliance with applicable personal data protection and the processing of personal data (GDPR lesgislation or Algemene vordering gegevens bescherming (EU vordering 2016/679))

Data are anonymous if no one, not even the researcher, can connect the data to the individual who provided it. No identifying information is collected from the individual.
When data are coded, there continues to be a link between the data and the individual who provided it. The research team is obligated to protect the data from disclosure outside the research according to the terms of the research protocol and the informed consent document. The subject's name or other identifiers should be stored separately from their research data and replaced with a unique code to create a new identity for the subject. Note that coded data are not anonymous.

## 10. Data Handling

Informed consent will be collected on paper. Pseudonymized data will be captured and stored at the REDCap and FatSecret platforms. FatSecret app will be used to capture 7-day food records.

Statistical analysis will be performed with pseudonymized data.

Special/sensitive categories of personal data will be recorded: medical history and genetic profile.

The project will obtain survey data (numeric and textual), experimental data (shotgun sequencing, mass spectrometry analysis, SCFA analyses, metabolomics, LPS and flagellin levels) and physical data (urine, blood and faecal samples). On the samples, experimental data will be generated in the form of laboratory analysis (numeric). Data generated through the use of apps and Redcap will be downloaded.

The data that will be generated above will be converted into spreadsheet (.txt, .sav, .csv, word) depending on the data type. This is particularly important since software that might be used could get outdated or a license might end. The files will be stored in a protected server from KU Leuven. A different file will include experimental codes to link experiments to the designated files. All generated data are pseudonymized with the key behind a personal password.

## 11. Data Management

The data will be stored on servers centrally managed by ICTS KU Leuven and with back-up capacities (KU Leuven enterprise box, Largevolumestorage) both during and after the research. If needed, extra storage capacity will be bought externally.

Pseudonymized data will be captured and stored at the REDCap and FatSecret platforms. All data collected during the trial will be pseudonymized before data processing and statistical analysis. Statistical analysis will be performed with R statistical software.

Data collection will be performed by the Dr. Judith Wellens, dietitians, and investigators. Data processing will be performed by the Dr. Judith Wellens. Statistical analysis will be performed by the Dr. Judith Wellens.

## 12. Translational research

Fecal samples will be collected for intestinal microbiota analysis and faecal calprotectin measurements. Urine will be collected during the lactulose mannitol test. Serum samples will be stored for proteomics analysis. The genetic profile of all included participants will be characterized using a custom-made assay (molecular inversion probes – MIP- genotyping assay) covering all 242 known loci, and a genetic risk score for all loci combined will be calculated.

All samples will be stored at the IBD Leuven biobank.

## 13. Publication Policy

Publications will be coordinated by the Principal Investigator. Authorship to publications will be determined in accordance with the requirements published by the International Committee of Medical Journal Editors and in accordance with the requirements of the respective medical journal.

## 14. Insurance/Indemnity

In accordance with the Belgian Law relating to experiments on human persons dated May 7, 2004, Sponsor shall assume, even without fault, the responsibility of any damages incurred by a Study Patient and linked directly or indirectly to the participation to the Study and shall provide compensation therefore through its insurance.

## 15. Financial Aspects

For this project the grant of prof. João Sabino will be used (#2002-04254) to cover the financial aspects.

## 16. References


1. Tuohy KM, Hinton DJS, Davies SJ, Crabbe MJC, Gibson GR, Ames JM. Metabolism of Maillard reaction products by the human gut microbiota - Implications for health. Mol Nutr Food Res. 2006;50(9):847–57.

2. Asioli D, Aschemann-Witzel J, Caputo V, Vecchio R, Annunziata A, Næs T, et al. Making sense of the "clean label" trends: A review of consumer food choice behavior and discussion of industry implications. Food Res Int [Internet]. 2017;99:58–71. Available from: http://dx.doi.org/10.1016/j.foodres.2017.07.022

3. Marion-Letellier R, Amamou A, Savoye G, Ghosh S. Inflammatory bowel diseases and food additives: To add fuel on the flames! Nutrients. 2019;11(5):1–12.

4. Monteiro CA, Moubarac JC, Cannon G, Ng SW, Popkin B. Ultra-processed products are becoming dominant in the global food system. Obes Rev. 2013;14(S2):21–8.

5. Seiquer I, Rubio LA, Peinado MJ, Delgado-Andrade C, Navarro MP. Maillard reaction products modulate gut microbiota composition in adolescents. Mol Nutr Food Res. 2014;58(7):1552–60.

6. Delgado-Andrade C, Seiquer I, Navarro MP, Morales FJ. Maillard reaction indicators in diets usually consumed by adolescent population. Mol Nutr Food Res. 2007;51(3):341–51.

7. van der Lugt T, Opperhuizen A, Bast A, Vrolijk MF. Dietary advanced glycation endproducts and the gastrointestinal tract. Nutrients. 2020;12(9):1–28.

8. Hofmann SM, Dong H, Li Z, Cai W, Altomonte J, Thung SN, et al. Restricted Intake of Dietary Glycoxidation Products in the db / db Mouse. Diabetes. 2002;51(July):2082–9.

9. Vlassara H, Cai W, Crandall J, Goldberg T, Oberstein R, Dardaine V, et al. Inflammatory mediators are induced by dietary glycotoxins, a major risk factor for diabetic angiopathy. Proc Natl Acad Sci U S A. 2002;99(24):15596–601.

10. Cai W, He JC, Zhu L, Peppa M, Lu C, Uribarri J, et al. High levels of dietary advanced glycation end products transform low-density lipoprotein into a potent redox-sensitive mitogen-activated protein kinase stimulant in diabetic patients. Circulation. 2004;110(3):285–91.

11. Sukino S, Nirengi S, Kawaguchi Y, Kotani K, Tsuzaki K, Okada H, et al. Effects of a Low Advanced Glycation End Products Diet on Insulin Levels: The Feasibility of a Crossover Comparison Test. J Clin Med Res. 2018;10(5):405–10.

12. Uribarri J, Woodruff S, Goodman S, Cai W, Chen XUE, Pyzik R, et al. AGE's in Foods and practical ways to reduce them. J Am Diet Assoc. 2010;110(6):911–6.

13. Zinöcker MK, Lindseth IA. The western diet–microbiome-host interaction and its role in metabolic disease. Nutrients. 2018;10(3):1–15.

14. Mills DJS, Tuohy KM, Booth J, Buck M, Crabbe MJC, Gibson GR, et al. Dietary glycated protein modulates the colonic microbiota towards a more detrimental composition in ulcerative

colitis patients and non-ulcerative colitis subjects. J Appl Microbiol. 2008;105(3):706–14.

15. Shangari N, Depeint F, Furrer R, Bruce WR, Popovic M, Zheng F, et al. A thermolyzed diet increases oxidative stress, plasma α-aldehydes and colonic inflammation in the rat. Chem Biol Interact. 2007;169(2):100–9.

16. Body-Malapel M, Djouina M, Waxin C, Langlois A, Gower-Rousseau C, Zerbib P, et al. The RAGE signaling pathway is involved in intestinal inflammation and represents a promising therapeutic target for Inflammatory Bowel Diseases. Mucosal Immunol [Internet]. 2019;12(2):468–78. Available from: http://dx.doi.org/10.1038/s41385-018-0119-z

17. Andrassy M, Igwe J, Autschbach F, Volz C, Remppis A, Neurath MF, et al. Posttranslationally modified proteins as mediators of sustained intestinal inflammation. Am J Pathol. 2006;169(4):1223–37.

18. Zen K, Chen CX-J, Chen Y-T, Wilton R, Liu Y. Receptor for Advanced Glycation Endproducts Mediates Neutrophil Migration across Intestinal Epithelium. J Immunol. 2007;178(4):2483–90.

19. Ciccocioppo R, Vanoli A, Klersy C, Imbesi V, Boccaccio V, Manca R, et al. Role of the advanced glycation end products receptor in Crohn’s disease inflammation. World J Gastroenterol. 2013;19(45):8269–81.

20. Zhang Q, Wang Y, Fu L. Dietary advanced glycation end-products: Perspectives linking food processing with health implications. Compr Rev Food Sci Food Saf. 2020;19(5):2559–87.

# Appendices

## Appendix 1: Questionnaire

1. **Name: ……………………………….**
2. **Date of birth: ………….**
3. Sex: ………….
4. Height: …………. cm
5. **Weight: …………. Kg**
6. **Ethnicity:**
    - White
    - Asian
    - Black, African or Caribbean
    - Mixed
    - Other: ………….
7. Smoking status:
    - Never smoked
    - Currently smoking
        - Type (cigarettes, cigars, vaping): ………….
        - Number/ day: ………….
    - Passive smoking

- Former smoker
  - Date stopped: ………….
  - Date started: ………….
  - Number of cigarette/cigars smoked (type + amount/day): ………….

8. **Intake of pain/anti-inflammatory medication**:
   - Last use of painkillers/anti-inflammatory medication: ………….
   - Type and frequency of:
     i. Aspirin: ………….
     ii. NSAID (Brufen, Ibuprofen, Gambaran,..): ………….
     iii. Paracetamol/Dafalgan: ………….

9. **Last episode of antibiotic, prebiotic or probiotic use**:
   - > 1 year ago
   - 6 months – 1 year ago
   - < 6 months ago
   - < 3 months ago

10. **Current medications or supplements if applicable (name, dosage, frequency). This includes contraceptive medication**.
    - ………….
    - ………….
    - ………….

11. Past medical and surgical history (disease/surgery and timing)
    - ………….
    - ………….
    - ………….

12. Personal history of inflammatory bowel disease (IBD)
    - Yes
    - No

13. Personal history of irritable bowel syndrome (IBS)
    - Yes
    - No

14. Personal history of any eating disorder:
    - Yes
    - No

15. Food allergies or intolerances, if yes, please specify which reaction to what type of food:
    - Yes: ………….
    - No

16. Family history of IBD: ………….

17. **Bristol stool type**: ………….

18. **Current abdominal complaints, please specify**: ………….

19. **Current general health complaints, please specify**: ………….

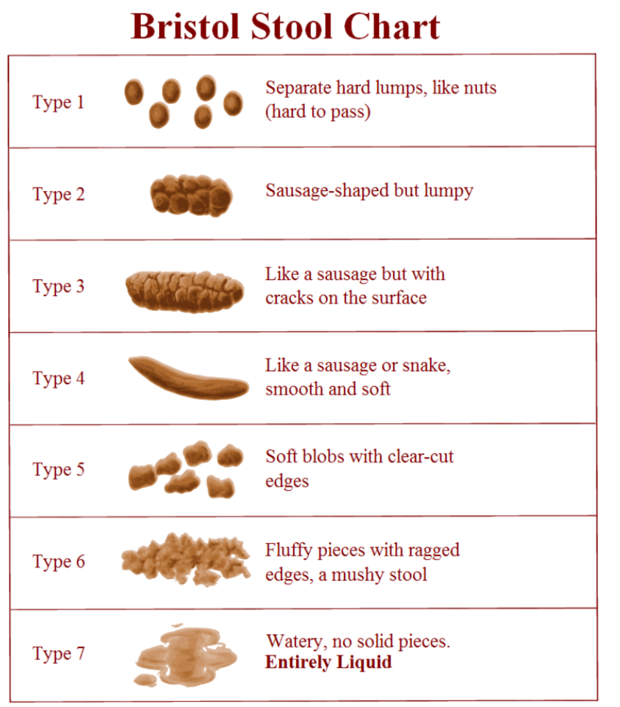


First published: Lewis SJ, Heaton KW (1997) Stool form scale as a useful guide to intestinal transit time. Scandinavian Jorunal of Gastroenterology 32: 920–4

Remark: questions in **bold** will be asked in the follow-up questionnaire (Redcap)

## Appendix 2: Menu

| Steeds toegestaan |
|---|
| Water |
| Fruitsap |
| Fruit |
| Melk |

| Niet toegestaan |
| --- |
| Roken |
| Alcohol |
| Koffie |
| Andere theesoorten dan aangegeven |
| Chocolade |
| Chips |
| Koeken, cakes, bisuits, koffiekoeken |
| Andere snacks dan aangegeven |

## High AGEs menu (gebakken, gegrild)

| | |
| --- | --- |
| Snacks (vrij te verdelen) | Bruscetta in oven |
| | Gebakken appel en perencrumble |
| | fruit |
| Drank | Zwarte thee |
| Opmerking | Toast met boter en beleg mag vervangen worden door croque monsieur |

| Dag 1 |
| --- |
| Granola met amandel, chiazaad en kokosschilvers (gebakken) |
| Toast met boter en beleg |
| Pikante Thaise curry |

| Dag 2 |
| --- |
| Granola met amandel, chiazaad en kokosschilvers (gebakken) |
| Toast met boter en beleg |
| Schelvis met courgette en aardappelen |

| Dag 3 |
| --- |
| Granola met amandel, chiazaad en kokosschilvers (gebakken) |
| Toast met boter en beleg |
| Zalm met groenten en dillesaus |

| Dag 4 |
| --- |
| Granola met amandel, chiazaad en kokosschilvers (gebakken) |
| Toast met boter en beleg |
| Noedels met kip |

| Dag 5 |
| --- |
| Granola met amandel, chiazaad en kokosschilvers (gebakken) |
| Toast met boter en beleg |
| Wraps met kip en bonen |

| Dag 6 |
| --- |
| Granola met amandel, chiazaad en kokosschilvers (gebakken) |
| Spiegelei (gebakken in boter) met toast |
| Kalkoen met gebakken wortelen en aardappelen |

| Dag 7 |
| --- |
| Granola met amandel, chiazaad en kokosschilvers (gebakken) |
| Omelet gebakken in boter en toast |
| Pasta met bloemkool en hamblokjes |

| Dag 8 |
| --- |
| Granola met walnoot en pompoenpitten (gebakken) |
| Toast met boter en beleg |
| Kabeljauw met prei en aardappelen |

| Dag 9 |
| --- |
| Granola met walnoot en pompoenpitten (gebakken) |
| Toast met boter en beleg |
| Kalkoen met gebakken aardappelen en broccoli |

| Dag 10 |
| --- |
| Granola met walnoot en pompoenpitten (gebakken) |

| |
|---|
| Toast met boter en beleg |
| Kabeljauw met groene asperges en kerstomaten |

| Dag 11 |
|---|
| Granola met walnoot en pompoenpitten (gebakken) |
| Toast met boter en beleg |
| Schotel met zoete aardappel en kip |

| Dag 12 |
|---|
| Granola met walnoot en pompoenpitten (gebakken) |
| Toast met boter en beleg |
| Wraps met kip en avocado |

| Dag 13 |
|---|
| Granola met walnoot en pompoenpitten (gebakken) |
| Spiegelei (gebakken in boter) met toast |
| Zoete rijstschotel met rundsgehakt |

| Dag 14 |
|---|
| Granola met walnoot en pompoenpitten (gebakken) |
| Omelet gebakken in boter en toast |
| Balletjes in tomatensaus |

## Low AGEs menu (rauw, gekookt, gestoomd)

| | |
|---|---|
| Snacks (vrij te verdelen) | Tomaat mozarella |
| | Ongebakken appel en perencrumble |
| | fruit |
| Drank | Zwarte thee |
| Opmerking | Brood met boter en beleg mag zowel kaas als hesp bevatten |

| Dag 1 |
|---|

| |
|---|
| Granola met amandel, chiazaad en kokosschilvers (ongebakken) |
| Brood met boter en beleg |
| Pikante Thaïse curry |

| Dag 2 |
|---|
| Granola met amandel, chiazaad en kokosschilvers (ongebakken) |
| Brood met boter en beleg |
| Schelvis met courgette en aardappelen |

| Dag 3 |
|---|
| Granola met amandel, chiazaad en kokosschilvers (ongebakken) |
| Brood met boter en beleg |
| Zalm met groenten en dillesaus |

| Dag 4 |
|---|
| Granola met amandel, chiazaad en kokosschilvers (ongebakken) |
| Brood met boter en beleg |
| Noedels met kip |

| Dag 5 |
|---|
| Granola met amandel, chiazaad en kokosschilvers (ongebakken) |
| Brood met boter en beleg |
| Wraps met kip en bonen |

| Dag 6 |
|---|
| Granola met amandel, chiazaad en kokosschilvers (ongebakken) |
| Zacht gekookt ei met brood en boter |
| Wortelpuree met kalkoen |

| Dag 7 |
|---|
| Granola met amandel, chiazaad en kokosschilvers (ongebakken) |
| Zacht- of hardgekookt ei met brood en boter |
| Pasta met bloemkool en hamblokjes |

| Dag 8 |
| --- |
| Granola met walnoot en pompoenpitten (gebakken) |
| Brood met boter en beleg |
| Kabeljauw met prei en aardappelen |

| Dag 9 |
| --- |
| Granola met walnoot en pompoenpitten (gebakken) |
| Brood met boter ene beleg |
| Broccolipuree met kalkoen |

| Dag 10 |
| --- |
| Granola met walnoot en pompoenpitten (gebakken) |
| Brood met boter en beleg |
| Kabeljauw met groene asperges en kerstomaten |

| Dag 11 |
| --- |
| Granola met walnoot en pompoenpitten (gebakken) |
| Brood met boter en beleg |
| Schotel met zoete aardappel en kip |

| Dag 12 |
| --- |
| Granola met walnoot en pompoenpitten (gebakken) |
| Brood met boter en beleg |
| Wraps met kip en avocado |

| Dag 13 |
| --- |
| Granola met walnoot en pompoenpitten (gebakken) |
| Zacht- of hardgekookt ei met brood en boter |
| Zoete rijstschotel met rundsgehakt |

| Dag 14 |
| --- |
| Granola met walnoot en pompoenpitten (gebakken) |
| Zacht- of hardgekookt ei met brood en boter |
| Balletjes met tomatensalade |

## Appendix 3: Recruitment flyer

### *GEZOCHT: GEZONDE VOLWASSENEN VOOR EEN STUDIE OVER GEZONDE VOEDING*

Ben jij een gezonde volwassene met interesse in gezonde voeding en een hart voor wetenschap? Lees dan zeker verder.

*Waarover gaat de studie?*

Deze studie bestudeert het effect van kookmethoden op algemene en darmgezondheid. Praktisch zullen 2 dieëten met elkaar vergeleken worden: de voeding bakken of grillen enerzijds, of koken of stomen anderzijds. Na het volgen van zo’n dieet worden ontstekingsparameters in bloed en stoelgang onderzocht, alsook veranderingen in het microbioom.

*Wie kan ik hiermee helpen?*

Op deze manier proberen wij nieuwe behandelingen te ontwikkelen voor patiënten met chronische darmaandoeningen. Natuurlijk kan de studie ook licht werpen op de effecten van voedselbereiding op gezonde volwassenen.

*Wat wordt er van mij verwacht?*

Je zal gevraagd worden 2 keer 2 weken een dieet te volgen, samengesteld door de arts-onderzoekers en diëtisten. Alle nodige voorzieningen voor deze studie, zoals het eten en de stoomkoker, zullen door het onderzoeksteam aangeboden worden.

Ook zal je gevraagd worden bloed-, urine- en stoelgangsstalen te doneren.

*Wanneer vindt de studie plaats?*

De studie gaat van start op 1 november 2021. Recrutering en screening zal echter in september plaatsvinden.

*Voor meer info of vragen:*

Judith.wellens@kuleuven.be

STEAMM

Study on Treating by Eating: role of AGEs on Mucosal barrier and Microbiome

Deze studie (S65600) werd goedgekeurd door het Ethische Commissie onderzoek UZ/KU Leuven op 17/12/2021.

## Appendix 4: Frequently asked questions

Beste deelnemer,

Eerst en vooral hartelijke dank om deel te nemen aan onze studie!

Omdat we weten dat er tijdens de contactmomenten zeer veel informatie gegeven wordt, vatten we hier nog even de belangrijkste zaken voor u samen.

Bij bijkomende vragen of problemen zijn wij ook steeds beschikbaar via mail of telefoon.

E-mail: judith.wellens@kuleuven.be

Tel: IBD labo 016/33.02.77 of 016/34.09.98 of DECT Judith Wellens: 016/34.69.28

Veel succes en eet smakelijk!

Het STEAMM-studie team.

STEAMM

Study on Treating by Eating: role of AGEs on Mucosal barrier and Microbiome

### *Doel van de studie:*

Via deze studie willen wij de effecten van voedselbereiding bestuderen op ontsteking in de darm en in het lichaam. Ook effecten op het microbioom (de bacteriën) in de darm zal bestudeerd worden. De resultaten kunnen ons in staat stellen betere voedingsadviezen te geven aan de algemene bevolking, maar in het bijzonder patiënten met chronische darmziekten.

### *Hoe verloopt de studie:*

Nadat u alle nodige informatie gekregen heeft van het researchteam en de geïnformeerde toestemming getekend heeft, kunnen we aan de slag.

Gedurende 1 week vragen wij u om nauwkeurig alle genuttigde voeding in te geven in een applicatie op uw smartphone: FatSecret. Deze app is gratis te downloaden en makkelijk in gebruik. Achteraf kunnen wij dan de data bekijken en ons een idee vormen van uw 'gewoonlijk' voedingspatroon. Op het einde van de week en net voor aanvang van de eigenlijke studie, lassen wij een moment in van staalname, om een basis voor u persoonlijk vast te leggen.

U wordt hiervoor verwacht nuchter naar UZ Leuven Gasthuisberg te komen voor een bloedafname en een urinetest (dit omvat het afstaan van een urinestaal, het drinken van een suikerdrank, waarna enkele uren later een tweede urinestaal gevraagd wordt). Ook vragen wij een stoelgangsstaal die je thuis hebt afgenomen mee te brengen in een speciale kit (die je van ons krijgt).

De urinetest zal enkele uren in beslag, dus misschien wilt u wat lectuur meebrengen voor tijdens het wachten.

De eigenlijke studie kan dan van start gaan en duurt 4 weken. Hiervan zal u gedurende 2 weken een dieet nuttigen waarbij de voeding gekookt/gestoomd wordt, en 2 weken waarbij deze gebakken/gegrild wordt. Het gebruik van FatSecret is ook tijdens deze periode noodzakelijk om studieresultaten achteraf te kunnen koppelen aan uw eetpatroon.

Na elk dieet van 2 weken volgt een moment van staalafname dat identiek zal verlopen als de eerste keer. Om uw veiligheid en gezondheid te garanderen zal de bloedname ook algemene testen bevatten die onder andere uw suiker- en vetmetabolisme controleren. Als er iets mis zou zijn, wordt u hiervan meteen op de hoogte gesteld. Al deze testen zijn kosteloos voor u.

Gedurende deze 4 weken, zullen de ingrediënten en recepten voor bereiding voor u voorzien worden, alsook een stoomkoker. Deze voedselpakketten kan u elke maandag en donderdag afhalen aan de Collect and Co van Colruyt Leuven. Op maandagen dient u ook een deel af te halen op de studiedienst.

Indien gebruik van FatSecret en staalname goed verloopt, krijgt u de stoomkoker van ons cadeau.

### *Wat mag ik eten?*

*Ontbijt*

Het ontbijt zal steeds bestaan uit granola, die voor u bereid werd door het studieteam. Yoghurt om hierbij te nuttigen zal ook voorzien worden.

*Middagmaal*

Het middagmaal zal een broodmaaltijd zijn, waarvoor beleg voorzien wordt. Beleg mag naar eigen smaak gecombineerd of gekozen worden zolang het afkomstig is uit het voedselpakket. U kiest ook zelf hoeveel brood u eet.

Omdat we weten dat brood op het einde van de week vaak niet meer zo lekker is, bent u vrij om vers brood te kopen als u dit prefereert.

*Avondeten*

Recepten voor elke dag worden voorzien alsook de ingrediënten die u hiervoor nodig heeft. Enkel kruiden dient u zelf te voorzien en kan u ook gebruiken naar eigen smaak.

*Snacks en tussendoortjes*

Snacks die steeds toegelaten zijn en voorzien zullen worden zijn fruit, bruscetta/tomaat mozarella en appel- en perencrumble.

Andere snacks zoals koekjes, chocolade, gebak, chips, koffiekoeken, snoep, kauwgom… zijn niet verenigbaar met de studie. U wordt dan ook gevraagd dit gedurende een maand niet te eten.

*Drank*

Er zal thee voorzien worden om te drinken tijdens de studie. Citroen, suiker en melk mogen toegevoegd worden naar smaak. Daarnaast bent u ook vrij om melk, water en fruitsap te drinken.

Alcoholische dranken, koffie en frisdranken zijn niet verenigbaar met de studie en u wordt gevraagd dit niet te drinken tijdens de studie. Ook andere theesoorten dan deze in uw pakket zijn niet toegelaten.

*Roken*

Roken is niet verenigbaar met de studie en u wordt gevraagd hiervan af te zien.

*Wat als ik nog honger heb?*

De menu's zijn samengesteld door diëtisten met oog voor voldoende nutritionele waarde en calorische inname. Ook zijn snacks voorzien moest u toch nog honger (of zin) hebben tussendoor.

*Wat als ik over heb? Moet ik alles opeten?*

Om deze reden vragen wij u FatSecret nauwkeurig in te vullen zodat wij weten hoeveel u gegeten hebt. Overschotten kunnen op een later moment gegeten worden (wraps als lunch bijvoorbeeld), maar mogen niet opnieuw opgewarmd worden.

*Wat als ik iets niet lust?*

Indien je een ingrediënt niet lust, mag je dit weglaten in beide gerechten (zowel in het gebakken als in het gestoomde menu). Als je bijvoorbeeld geen koriander op je vis wil, laat je dit steeds weg.

*Wat als ik ziek word/medicatie moet nemen?*

In dit geval vragen wij u meteen contact op te nemen met het studieteam voor gepast advies.
